# SGLT2 inhibitors: mechanisms, physiology and pharmacology, and clinical applications

**DOI:** 10.1186/s43556-026-00604-3

**Published:** 2026-09-30

**Authors:** Xue-Ting Zheng, Hui-Yi Xie, Xiu-Heng Wang, Yue Zhao, Jia-Yan Yang, Yun-Xi Liu, Xian-Liang Yuan, Hui-Fang Tang, Zhi-Sheng Jiang, Heng-Jing Hu

**Affiliations:** 1 The First Affiliated Hospital, Department of Cardiovascular Medicine, Hengyang Medical School, University of South China, Hengyang, Hunan 421001 P.R. China; 2 The First Affiliated Hospital, Department of Endocrinology, Hengyang Medical School, University of South China, Hengyang, Hunan 421001 P.R. China; 3 Department of Cardiovascular Disease and Key Laboratory for Atherosclerosis of Hunan Province, Hengyang Medical School, University of South China, Hengyang, Hunan 421001 P.R. China

**Keywords:** SGLT2 inhibitors, Cardio-renal-metabolic continuum, Heart failure, Chronic kidney disease, Endothelial-to-mesenchymal transition, Precision medicine

## Abstract

Sodium-glucose cotransporter 2 (SGLT2) is a low-affinity, high-capacity transporter expressed predominantly in the renal proximal tubule, where it mediates the sodium-coupled reabsorption of most filtered glucose. SGLT2 inhibitors have evolved from glucose-lowering agents into disease-modifying therapies across the cardio-renal-metabolic continuum. Blockade of proximal tubular transport induces glycosuria, osmotic diuresis, restoration of tubuloglomerular feedback, resetting of renal haemodynamics, and altered fluid compartmentalisation. These early renal and circulatory effects interact with systemic metabolic adaptations, including pseudo-starvation signalling, erythropoiesis, altered uric acid handling, and changes in substrate availability. At the cellular level, SGLT2 inhibition has been linked to improved ionic homeostasis and mitochondrial quality control, reduced oxidative stress, modulation of inflammation, and attenuation of maladaptive tissue remodelling. Endothelial injury may connect these cellular effects with microvascular dysfunction and fibrosis. Endothelial dysfunction and endothelial-to-mesenchymal transition (EndMT) offer a mechanistic framework for understanding the relationship between microvascular injury and fibrosis. However, EndMT is best viewed as one component of a broader endothelial–stromal stress response rather than as a single proven mediator of clinical benefit. These pharmacodynamic and cellular effects help explain the consistent clinical efficacy of SGLT2 inhibitors across type 2 diabetes, chronic kidney disease, and heart failure (HF), while also informing emerging applications in acute HF, after myocardial infarction, and in patients with arrhythmia or metabolic dysfunction-associated steatohepatitis. This Review integrates mechanistic, pharmacological, and clinical evidence to identify unresolved translational gaps and future directions, including mechanism-informed biomarkers, imaging, spatial multi-omics, phenotype-guided patient stratification, dual sodium-glucose cotransporter 1/2 inhibition, and rational combination therapy.

## Introduction

Sodium-glucose cotransporter 2 (SGLT2) is a low-affinity, high-capacity transporter expressed predominantly in the S1 and S2 segments of the renal proximal tubule, where it mediates the sodium-coupled reabsorption of approximately 90% of filtered glucose [1, 2]. By coupling glucose transport to the transmembrane sodium gradient, SGLT2 serves as a central regulator of proximal tubular glucose handling and an accessible pharmacological target. The pharmacological origins of SGLT2 inhibition can be traced to phlorizin, a naturally occurring glucoside that established the feasibility of lowering blood glucose by inhibiting renal glucose transport. Modern synthetic SGLT2 inhibitors were developed to overcome the poor oral stability and limited transporter selectivity of early compounds, enabling targeted blockade of proximal tubular glucose reabsorption and therapeutic glycosuria in patients with type 2 diabetes mellitus (T2DM) [1, 2]. Their initial clinical role therefore centred on glucose lowering, with additional effects on body weight and blood pressure. Over the past decade, however, cardiovascular and kidney outcome trials have substantially broadened this therapeutic profile. Reductions in hospitalisation for heart failure (HF), slower progression of chronic kidney disease (CKD), and clinical benefits in patients without diabetes indicate that SGLT2 inhibitors act beyond glycaemic control [3–5]. This dissociation between glucose lowering and organ protection has repositioned SGLT2 inhibitors within the cardio-renal-metabolic continuum, a framework reflecting the frequent overlap of cardiovascular, kidney, and metabolic disorders in contemporary patient populations [6–8].

The expanding use of SGLT2 inhibitors has also revealed a mechanistic gap. Clinical benefits are now observed across T2DM, CKD, HF, and selected high-risk acute or post-acute settings, yet the pathways linking proximal tubular transporter inhibition to systemic organ protection remain incompletely integrated [4, 5, 9]. Mechanistic explanations centred on glycosuria, osmotic diuresis, natriuresis, ketone availability, anti-inflammatory effects, or antifibrotic signalling each capture part of the pharmacological response, but no single mechanism accounts for the breadth, timing, and context dependence of the benefits of SGLT2 inhibitors [10–12]. A cross-scale synthesis is therefore required to link renal transport inhibition to haemodynamic unloading, fluid redistribution, nutrient-sensing adaptation, mitochondrial and ionic stress responses, inflammatory modulation, and tissue remodelling. Endothelial homeostasis represents a key interface between these systemic adaptations and tissue remodelling because endothelial injury can translate metabolic and inflammatory stress into microvascular dysfunction and profibrotic signalling. Within this network, endothelial-to-mesenchymal transition (EndMT) offers a useful framework for understanding the links among endothelial injury, inflammatory amplification, and fibrotic remodelling. This Review integrates evidence on the mechanisms, physiology, pharmacology, clinical applications, and translational challenges of SGLT2 inhibition across the cardio-renal-metabolic continuum.

A coherent interpretation of SGLT2 inhibition must begin with the renal transporter but extend beyond it. Inhibition of SGLT2-mediated glucose and sodium transport in the proximal tubule initiates the pharmacological response, whereas clinically relevant consequences unfold through the resetting of renal haemodynamics, systemic fluid redistribution, metabolic adaptation, and cellular stress responses. These mechanistic layers also inform the interpretation of transporter selectivity, exposure–response relationships, pharmacodynamic boundaries, and safety considerations. Clinical evidence is therefore considered in the disease contexts in which these mechanisms are most relevant, including T2DM, CKD, HF across the ejection fraction spectrum, and selected emerging indications. The Review then addresses the remaining translational challenge: linking outcome benefits to tissue-level mechanisms, identifying responsive patient phenotypes, and designing dual-inhibition or combination strategies guided by biology rather than by disease labels alone.

## Mechanisms of action of SGLT2 inhibitors

**Fig. 1:**
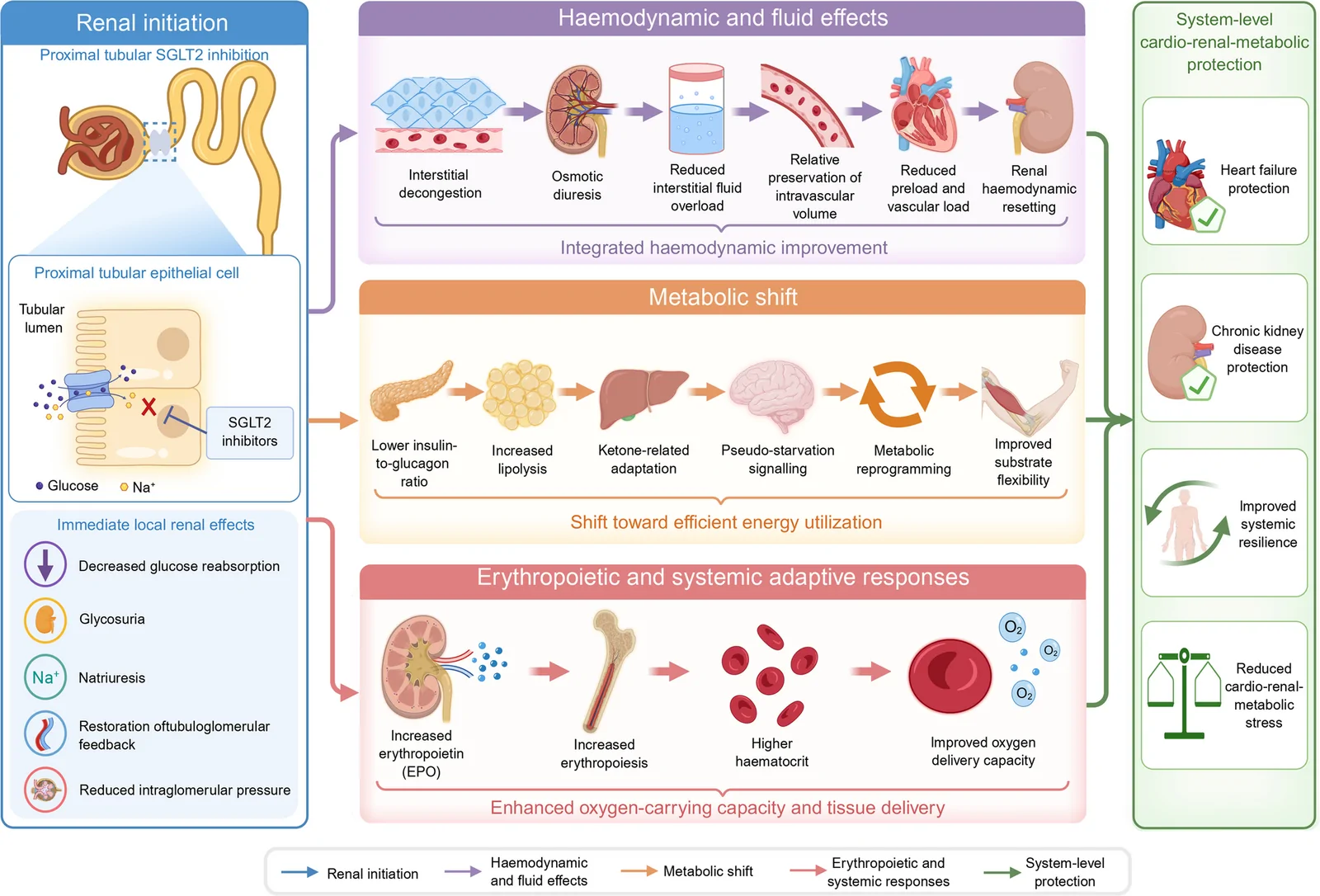
Systemic cardio-renal-metabolic reprogramming initiated by proximal tubular SGLT2 inhibition. Inhibition of SGLT2 in proximal tubular epithelial cells alters proximal glucose–sodium cotransport, inducing glycosuria, natriuresis, restoration of tubuloglomerular feedback and attenuation of maladaptive intraglomerular hypertension. These renal effects extend beyond the nephron through coordinated changes in fluid compartmentalisation, metabolic adaptation and oxygen-handling capacity. Glycosuria-driven osmotic diuresis favours interstitial decongestion while relatively preserving intravascular volume, thereby reducing preload and vascular load. Sustained urinary glucose loss lowers the insulin-to-glucagon ratio and favours lipolysis, ketone-related adaptation, pseudo-starvation signalling and broader metabolic reprogramming. Increased erythropoietin signalling and erythropoiesis raise haematocrit and may improve oxygen delivery to stressed tissues. These integrated responses link proximal tubular SGLT2 blockade to heart failure and chronic kidney disease protection, improved systemic resilience and reduced cardio-renal-metabolic stress. Abbreviations: SGLT2, sodium-glucose cotransporter 2

SGLT2 inhibitors lower blood glucose by blocking proximal tubular transport and reduce cardio-renal-metabolic risk across multiple clinical settings. Rather than acting solely as antihyperglycaemic agents, these drugs initiate a series of linked physiological responses that begin in the proximal tubule and extend to renal microvascular tone, systemic fluid handling, and cellular energy signalling. Interpreting the dissociation between their clinical benefits and baseline glycaemic status therefore requires a broader view of their pharmacological effects. The effects of proximal tubular transporter inhibition on cardio-renal outcomes may be amplified through tubuloglomerular feedback (TGF), haemodynamic unloading, and metabolic adaptation (Fig. 1).

### Classical renal mechanisms: glycosuria, natriuresis, and TGF

SGLT2 inhibitors are conventionally understood to act through glycosuria and natriuresis following inhibition of proximal tubular transport. Human kidney transcriptomic and single-cell data support the localisation of SGLT2/SLC5A2 to the proximal tubule, while suggesting that responses to SGLT2 inhibitors may extend beyond the transporter-expressing segment [13]. However, clinical profiling in acute HF questions whether sodium excretion is the primary determinant of early fluid shifts. Under these conditions, SGLT2 inhibition promotes osmotic diuresis primarily by increasing fractional glucose excretion, whereas fractional sodium excretion and urine osmolality remain relatively stable [14]. These findings suggest that the clinically relevant volume effects of SGLT2 inhibitors, including reductions in estimated extracellular volume and fluid overload without corresponding fat mass loss, may be mediated partly by electrolyte-sparing osmotic diuresis rather than conventional natriuresis [14–16].

Restoration of TGF may provide an important link between altered proximal tubular transport and kidney protection. In the hyperfiltering diabetic kidney, increased proximal reabsorption reduces sodium chloride delivery to the macula densa and decreases afferent arteriolar tone. SGLT2 blockade increases solute delivery to this distal sensor and may augment local adenosine signalling. High-resolution intravital multiphoton imaging in mice with type 1 diabetes showed that empagliflozin acutely constricted the afferent arteriole and normalised the single-nephron glomerular filtration rate (SNGFR) [17]. Related experimental studies have associated renoprotection during empagliflozin treatment with activation of TGF-related pathways and attenuation of complement-related injury signals, suggesting that the renal response involves more than haemodynamic changes alone [18]. This TGF response appears to depend substantially on A1 adenosine receptor signalling and may be reflected clinically by the characteristic initial “dip” in estimated GFR [17–19]. These changes in renal haemodynamics may persist despite physiological variation, including fluctuations in dietary protein intake that would otherwise alter renal perfusion [19]. Post hoc clinical data from individuals with youth-onset type 2 diabetes further suggest that empagliflozin may attenuate glomerular hyperfiltration and albuminuria, indicating that this mechanism may also be relevant beyond models of adult diabetic kidney disease [20].

Nevertheless, a model based solely on afferent arteriolar constriction does not capture the complexity of renal microvascular regulation. The macula densa integrates metabolic signals through parallel pathways that regulate vasomotor tone. For example, luminal glucose uptake through local macula densa SGLT1 transporters directly upregulates neuronal nitric oxide synthase 1 (NOS1). The resulting nitric oxide signal promotes vasodilation, suppresses TGF, and may contribute to early diabetic hyperfiltration [21]. Consequently, the microvascular response to SGLT2 blockade varies by context. Although afferent constriction is prominent in models of type 1 diabetes, haemodynamic studies in humans with type 2 diabetes suggest a broader vasodilatory response, with reductions in both pre- and post-glomerular vascular resistance [22].

The microvascular response may also differ during combination therapy. Longitudinal in vivo imaging showed that combined administration of SGLT2 inhibitors and ACE inhibitors reduced SNGFR without measurable changes in afferent or efferent arteriolar diameter, suggesting that other mechanisms may contribute to the reduction [23]. Similarly, a randomised crossover trial in people with type 2 diabetes suggested that SGLT2 inhibition and renin–angiotensin system blockade may have additive or complementary effects on renal haemodynamics [22]. Collectively, these findings suggest that SGLT2 blockade may attenuate maladaptive intraglomerular hypertension through context-dependent changes in renal microvascular function. This shared functional endpoint may link inhibition of proximal tubular transport to broader haemodynamic effects [17, 19, 22, 23].

### Haemodynamic and volume-related effects

The systemic haemodynamic effects of SGLT2 inhibition differ from those of conventional loop diuretics. This distinction may help explain why SGLT2 inhibitors can promote circulatory unloading with less abrupt intravascular volume contraction, electrolyte disturbance, or neurohormonal activation than aggressive loop-diuretic escalation. Their haemodynamic effects may therefore reflect changes in fluid compartmentalisation and vascular capacitance rather than diuresis alone [14, 24, 25].

Clinical studies of fluid dynamics suggest that SGLT2 inhibition can reduce extracellular volume and interstitial fluid overload while having a more modest effect on intravascular plasma volume [15, 16, 24]. This pattern may partly reflect electrolyte-sparing osmotic diuresis. In acute HF cohorts, empagliflozin increased fractional glucose excretion and plasma osmolality without a clear corresponding increase in fractional sodium excretion or urine osmolality [14]. The resulting changes in osmotic gradients may promote fluid movement from the interstitium into the vascular space. This mechanism could reduce tissue oedema, consistent with reductions in bioimpedance-derived excess extracellular water without corresponding fat mass loss, while limiting arterial underfilling [16].

These localised fluid shifts may contribute to rapid haemodynamic and symptomatic improvement. Echocardiographic assessment showed that empagliflozin reduced left ventricular filling pressure, as reflected by an improved E/e′ ratio, within 24 h of administration [26]. This change occurred without a reduction in cardiac index or alteration in systemic vascular resistance [26]. Invasive haemodynamic data qualify these findings: empagliflozin reduced resting pulmonary capillary wedge pressure in patients with type 2 diabetes at high cardiovascular risk, but the reduction during submaximal exercise was not clearly greater than that observed with placebo [27]. Continuous invasive monitoring with CardioMEMS sensors also showed a sustained reduction in pulmonary artery diastolic pressure during empagliflozin treatment [28]. This pulmonary decongestive effect appeared to increase over time and occurred without clear corresponding changes in loop-diuretic dosing, suggesting that volume depletion alone may not fully explain the observed haemodynamic changes [28].

Changes in venous capacitance and effective circulating volume may also be involved. During SGLT2 inhibitor therapy, estimated total blood volume decreases only modestly, whereas estimated stressed blood volume (SBV), the functional compartment that generates vascular pressure, decreases to a greater extent. This reduction in SBV is associated with lower pulmonary capillary wedge pressure [25]. This pattern is consistent with redistribution of blood from the stressed to the unstressed venous compartment and suggests possible effects on venous capacitance, sympathetic tone, or vascular volume partitioning, although the relative contribution of each mechanism remains uncertain [25, 29].

Haemoconcentration may also reflect more than plasma volume contraction. The persistent increase in haematocrit may not result solely from diuresis. Empagliflozin treatment is associated with a transient early increase in renal erythropoietin secretion, followed by changes in erythropoiesis, red blood cell morphology, and iron-store markers [30]. Studies of dapagliflozin and empagliflozin likewise suggest that SGLT2 inhibition may affect erythropoiesis and iron handling, including hepcidin suppression and delayed increases in red blood cell indices [31, 32]. Thus, rather than simply indicating reduced plasma volume, these haematological changes may also reflect altered erythropoiesis and could increase oxygen-carrying capacity. Overall, the circulatory effects of SGLT2 inhibition may involve modest interstitial decongestion, redistribution of venous volume, and changes in oxygen transport. Each may contribute to lower haemodynamic stress without substantial sodium-rich diuresis [16, 25, 30, 31, 33].

### Metabolic shift and pseudo-starvation signalling

Haemodynamic unloading and improved oxygen transport may alter the metabolic environment of stressed organs. SGLT2 inhibition also affects energy balance by increasing urinary glucose excretion (UGE) and producing persistent, mild caloric loss. In heart failure with preserved ejection fraction (HFpEF), dapagliflozin reduced resting and exercise pulmonary capillary wedge pressure and plasma volume. It also lowered arterial lactate during low-level exercise without changing oxygen consumption or cardiac output, suggesting that haemodynamic and metabolic responses may occur concurrently in vivo [34]. This metabolic state differs from both true starvation and improved glycaemic control alone. It may instead represent a mild nutritional perturbation that modifies substrate availability, redox balance, and nutrient-sensing pathways in tissues affected by diabetes, obesity, HF, or CKD [29, 34–36].

The “thrifty substrate” hypothesis proposes that the cardiac effects of SGLT2 inhibition may partly reflect a shift towards ketone body utilisation [37]. Persistent glycosuria during SGLT2 blockade may reduce the insulin-to-glucagon ratio, thereby favouring lipolysis and hepatic ketogenesis. In nondiabetic porcine models of HF, empagliflozin shifted myocardial fuel utilisation away from anaerobic glycolysis towards free fatty acids, branched-chain amino acids, and ketone bodies [38]. Because ketone oxidation may be energetically favourable under selected conditions, this substrate shift has been proposed to improve myocardial efficiency, attenuate adverse left ventricular remodelling, and support systolic function [38]. In a nondiabetic porcine model of acute myocardial infarction, both empagliflozin pretreatment and intravenous β-hydroxybutyrate infusion were associated with reduced infarct size, less microvascular obstruction, and greater myocardial salvage, suggesting that ketone availability may be relevant during acute ischaemia–reperfusion stress [39]. Post hoc clinical data following acute myocardial infarction have also shown associations between β-hydroxybutyrate dynamics and cardiac imaging markers. However, these observations remain hypothesis-generating and do not establish a causal ketone-mediated mechanism [40].

The metabolic effects of SGLT2 inhibition cannot be adequately explained by a single-substrate model. Metabolomic studies across the HF spectrum suggest that clinically significant ketosis is uncommon during SGLT2 inhibitor treatment. In HFrEF, dapagliflozin increased ketone-related metabolites and short- to medium-chain acylcarnitines, but the absolute increase in ketones was modest and supraphysiological concentrations were not observed [41]. In HFpEF, the PRESERVED-HF metabolomic analysis found no significant dapagliflozin-associated changes in circulating metabolite clusters representing ketone, fatty acid, or branched-chain amino acid pathways [42]. A pooled analysis across the ejection fraction spectrum showed relatively consistent changes in short- and medium-chain acylcarnitines, whereas ketone-related changes became less pronounced at higher left ventricular ejection fractions [43]. The EMPA-VISION trial found no improvement in bulk myocardial energetics, assessed using the phosphocreatine-to-ATP ratio by magnetic resonance spectroscopy, after 12 weeks of empagliflozin treatment in either HFrEF or HFpEF [44]. In stable chronic HF, empagliflozin increased β-hydroxybutyrate, but changes in ketone levels did not show a simple relationship with vascular outcomes [45]. These findings do not exclude metabolic effects but argue against ketone availability as a universal explanation for the efficacy of SGLT2 inhibitors.

Pseudo-starvation signalling provides one framework for reconciling these findings. Rather than solely increasing substrate supply, SGLT2 inhibitors may reduce nutrient-surplus signalling and activate adaptive stress pathways that share features with fasting. Multi-organ metabolic flux analysis showed that dapagliflozin altered glucose oxidation and nutrient signalling across the kidney, liver, and heart rather than uniformly restoring glycolysis. AMPK activation and mechanistic target of rapamycin complex 1 (mTORC1) inhibition were recurring features [35]. Across nephron compartments, exposure to SGLT2 inhibitors has been associated with attenuation of diabetes-related tubular metabolic disturbances and mTORC1 activity [13]. In experimental diabetic kidney disease, β-hydroxybutyrate has been reported to act both as an energy substrate and as a signalling metabolite. Its effects have been associated with reduced pathological mTORC1 activation, restoration of impaired renal lipolysis, and protection of proximal tubular cells and podocytes [46]. Conceptual models of fasting mimicry also place sirtuin 1 (SIRT1)/AMPK activation, mTOR inhibition, and autophagic maintenance within an adaptive stress response that may increase cellular resistance to stress. However, this framework is not yet fully established [36].

Pseudo-starvation signalling may also interact with systemic lipid handling. In models of hepatic steatosis, dapagliflozin-associated AMPK activation and mTORC1 suppression were accompanied by restored autophagic flux, enhanced clearance of intracellular lipid droplets, and increased expression of fatty acid oxidation markers [47]. In vivo metabolic flux analyses suggest that SGLT2 inhibitors may increase glucose oxidation through the tricarboxylic acid (TCA) cycle in the kidney, liver, and heart, even when impaired glycolysis is not fully rescued [35]. Metabolomic analyses have also identified long-chain acylcarnitines, markers of incomplete fatty acid oxidation and mitochondrial stress, as correlates of worse HF-related health status [41–43]. Reductions in these adverse metabolic signatures may therefore be as relevant as increases in ketone levels.

Overall, the metabolic response to SGLT2 inhibition extends beyond substrate selection. SGLT2 inhibitors may reduce nutrient-surplus signalling, increase substrate flexibility, and engage stress-adaptive pathways that overlap with fasting biology. Ketone metabolism may contribute in selected contexts, particularly in HFrEF, acute injury models, or renal metabolic stress, but is unlikely to represent the sole underlying mechanism [39, 41, 42, 44, 46]. The metabolic effects of SGLT2 inhibition may therefore depend more on broader changes in nutrient-sensing pathways than on the preferential use of a single substrate. These changes may attenuate maladaptive hypertrophic signalling, support autophagic maintenance, and increase cellular tolerance to metabolic stress. The variable magnitude of ketone-related changes across settings is consistent with flexible metabolic adaptation rather than a universal shift to ketone-based energy production [29, 35, 36, 46, 47].

### Ionic homeostasis, mitochondrial protection, and oxidative stress

Adaptive metabolic responses may also depend on the ability of stressed cells to maintain ionic gradients and limit oxidative injury. In cardiomyocytes and cardiac microvascular endothelial cells, nutrient-sensing pathways, mitochondrial redox balance, and transmembrane ion flux are closely interconnected. Excess intracellular Na^+^ and secondary Ca^2+^ loading can impair relaxation, disrupt mitochondrial function, and enhance ROS–CaMKII signalling. Several experimental studies implicate altered ion handling in the cellular effects associated with SGLT2 inhibitors, although the relevant pathways cannot be fully explained by canonical renal SGLT2 blockade. Empagliflozin has been shown to reduce cytosolic Na^+^ and Ca^2+^ in isolated cardiomyocytes, partly through inhibition of Na^+^/H^+^ exchanger 1 (NHE1), while increasing mitochondrial Ca^2+^ availability [48]. In a pressure-overload/mineralocorticoid-induced model of HF, empagliflozin preserved cardiac function and reduced hypertrophy, fibrosis, oxidative stress, Na^+^/Ca^2+^ exchanger expression, and CaMKII activation, even in SGLT2-deficient mice. These findings suggest a possible SGLT2-independent pathway involving NHE1 and nitric oxide [49]. Although these data do not establish NHE1 as the predominant mechanism in patients, they suggest that ionic homeostasis may contribute to the cardiac effects of SGLT2 inhibition under metabolic stress.

Pathological Na^+^ entry may also involve the cardiac sodium channel. Oxidative stress enhances the late sodium current (late-INa), contributing to Na^+^ overload, impaired Ca^2+^ handling, and arrhythmogenic prolongation of the action potential. Empagliflozin, dapagliflozin, and canagliflozin inhibit H_2_O_2_-induced late-INa, and modelling studies suggest that empagliflozin may interact with a Nav1.5 region that overlaps the binding sites of established sodium-channel inhibitors [50]. However, these effects may not result solely from direct channel blockade. In a murine HFpEF model, prolonged empagliflozin exposure was required to reverse late-INa upregulation and action potential instability, and this response coincided with CaMKII inhibition [51]. Findings in human atrial cardiomyocytes from patients with HFpEF provide translational support: Na^+^ influx, late-INa, CaMKII activity, and CaMKII-dependent Nav1.5 phosphorylation were increased, whereas empagliflozin reduced Na^+^ influx and late-INa [52]. Thus, SGLT2 inhibition may modulate Na^+^ handling through NHE1 modulation, late-INa suppression, and reduced CaMKII-dependent channel remodelling, with the relative contribution of each pathway likely varying by disease state, cell type, and duration of drug exposure.

Mitochondrial stress may develop downstream of these ionic disturbances. Na^+^ and Ca^2+^ overload can increase mitochondrial stress, promote reactive oxygen species (ROS) generation, and sustain CaMKII-dependent injury pathways. In failing rat hearts, chronic dapagliflozin treatment shifted substrate oxidation towards ketone utilisation and was associated with improvements in mitochondrial redox state and systolic function and with reduced lipid peroxidation [53]. These findings suggest an association between substrate metabolism and redox status but do not establish ketone oxidation as the sole mechanism underlying the observed effects. Similar interactions between ion handling and redox regulation have been observed in the microvasculature. Dapagliflozin attenuated endothelial dysfunction following ischaemia/reperfusion in association with reduced xanthine oxidase activation, sarcoplasmic/endoplasmic reticulum Ca^2+^-ATPase 2 (SERCA2) oxidation, cytosolic Ca^2+^ overload, and CaMKII-cofilin signalling, together with preservation of cytoskeletal and barrier integrity [54]. Empagliflozin has also been reported to preserve cardiac microvascular endothelial cell function by limiting pathological mitochondrial fission through the DNA-PKcs/Fis1 axis and activating AMPKα1/ULK1/FUNDC1-dependent mitophagy, which may facilitate the removal of damaged mitochondria during reperfusion stress [55, 56]. These studies suggest that SGLT2 inhibitors may affect both cardiomyocytes and endothelial cells, with mitochondrial quality control potentially contributing to the maintenance of perfusion and barrier function.

Redox responses may differ among individual agents. Canagliflozin, which has greater affinity for SGLT1, reduced NADPH oxidase-derived superoxide and improved nitric oxide synthase coupling in human atrial myocardium. These effects were associated with an SGLT1-AMPK-Rac1 pathway and increased tetrahydrobiopterin availability [57]. This finding should not be extrapolated to all SGLT2 inhibitors because empagliflozin did not reproduce the same ex vivo redox profile in that study [57]. Other in vitro studies suggest that dapagliflozin may reduce ROS production and inflammatory stress through PI3K/AKT-related signalling in cardiomyocytes and endothelial cells, although these findings remain limited to cell models [58]. These ionic and mitochondrial effects may also be relevant to electrical stability. In an acute rat model of ischaemia/reperfusion, empagliflozin reduced reperfusion-induced ventricular arrhythmias and sudden cardiac death in association with ERK1/2-related survival signalling [59]. Across these models, SGLT2 inhibitor treatment has been associated with modulation of stress-sensitive Na^+^ entry, Ca^2+^ handling, mitochondrial quality control, and ROS production. The contribution of each pathway to clinical outcomes in humans remains uncertain.

### Anti-inflammatory, anti-oxidative, and anti-fibrotic effects

Ionic and oxidative stress may contribute to tissue injury by promoting sterile inflammation and maladaptive repair. Immune modulation may contribute to the cardiac effects observed with SGLT2 inhibitors, but these agents are not conventional broad-spectrum immunosuppressants. In experimental HF models, single-cell transcriptomic analyses suggest that dapagliflozin preferentially modifies cardiac macrophage-fibroblast crosstalk rather than acting as a direct fibroblast inhibitor. Similar changes have been observed in murine models with global deletion of the target transporter [60]. Related findings have been reported in a model of dyslipidaemia-induced diastolic dysfunction, in which metabolic stress activated inflammatory cardiac macrophage states, whereas dapagliflozin treatment was associated with a less inflammatory cardiac immune-infiltrate profile [61]. Together, these findings suggest that gliflozins may modulate local metabolic and inflammatory conditions rather than broadly suppress immune activity. This interpretation is consistent with differing clinical findings. Dapagliflozin treatment for 12 months was associated with reduced circulating interleukin-1 beta (IL-1β) and preserved mitochondrial respiration in peripheral immune cells, whereas empagliflozin did not significantly alter the post-infarction decline in global acute-phase reactants, including high-sensitivity C-reactive protein (hsCRP) and interleukin-6 (IL-6) [62, 63]. This pattern is more consistent with modulation of chronic, low-grade microenvironmental inflammation than with broad suppression of the acute inflammatory response.

These immunomodulatory effects may be linked to intracellular metabolic changes. The SGLT2 inhibitor-associated increase in β-hydroxybutyrate, together with reduced insulin levels, may attenuate signals that activate the NOD-like receptor family pyrin domain-containing 3 (NLRP3) inflammasome rather than produce nonspecific suppression of inflammation. In patients with type 2 diabetes and high cardiovascular risk, SGLT2 inhibition reduced IL-1β secretion from stimulated human macrophages more than sulfonylurea treatment despite comparable glucose lowering. These changes were accompanied by increased β-hydroxybutyrate and reduced insulin, glucose, and uric acid levels [64]. In fibrotic renal tissue, dapagliflozin altered TCA cycle-related metabolism and increased itaconate, an endogenous immunomodulatory metabolite that can suppress NLRP3 activation [65]. These metabolic effects may also extend to disease-specific uric acid handling and associated profibrotic stress. In hyperuricaemic nephropathy, dapagliflozin was associated with activation of the estrogen-related receptor alpha (ERRα)-organic anion transporter 1 (OAT1) axis, increased uric acid excretion, and reductions in urate-induced tubular injury, mitochondrial dysfunction, and tubulointerstitial fibrosis [66].

Persistent microenvironmental stress may promote cellular senescence, a recognised pathological feature of HFpEF. Gliflozins may affect senescence-associated transcriptional pathways upstream of cytokine production. Proteomic mapping in an experimental HFpEF model showed that empagliflozin treatment was associated with suppression of interferon-gamma-related STAT1-STING signalling, reduced expression of senescence markers such as p16 and p21, and attenuation of inflammatory cardiac remodelling [67]. Context-dependent effects have also been reported in other disease models. In a mouse model of arrhythmogenic cardiomyopathy, dapagliflozin restored hypoxia-inducible factor 2 alpha (HIF-2α) signalling and attenuated fibrofatty replacement, cardiac inflammation, and fibrotic remodelling [68]. In doxorubicin-induced cardiotoxicity, empagliflozin reduced fibrosis, apoptosis, and ferroptosis in association with lower NLRP3/MyD88/NF-κB-related inflammatory signalling [69]. In experimental autoimmune myocarditis, canagliflozin attenuated cardiac inflammation, NLRP3 inflammasome activation, and Th17-associated immune infiltration [70]. These findings support potential effects of SGLT2 inhibitors on inflammatory and remodelling pathways, although most of the evidence remains derived from animal or cell-based models.

Modulation of inflammatory and senescence-related pathways does not necessarily imply regression of established fibrosis. Available evidence suggests a distinction between changes in molecular markers and structural remodelling. Molecular findings are consistent with possible antifibrotic activity, but structural evidence remains limited. In the EMPEROR trials, empagliflozin was associated with reductions in circulating markers of type I and type III collagen turnover, including procollagen type I carboxy-terminal propeptide (PICP) and PRO-C3 peptides [71]. Urinary proteomic analyses have likewise identified changes in pathways related to collagen turnover, wound healing, and inflammation in diabetic kidney disease [72]. However, these biochemical and proteomic changes do not necessarily correspond to short-term improvement on cardiac imaging. A 12-month placebo-controlled cardiac magnetic resonance (CMR) trial found no significant improvement in myocardial extracellular volume fraction (ECV) or global strain despite evidence of systemic inflammatory modulation [62]. The absence of detectable imaging changes does not exclude biological effects on fibrosis but limits structural inferences based on short-term biomarker changes. SGLT2 inhibitors may more readily attenuate active metabolic and inflammatory signals associated with new matrix deposition than reverse established, cross-linked collagen. Detecting anatomical reverse remodelling in the failing human heart may therefore require longer follow-up, selection of populations with active fibrotic turnover, or combination therapies that directly promote matrix resolution.

### Cellular and molecular mechanisms of tissue protection: endothelial homeostasis, fibrotic remodelling, and EndMT

Inflammatory and fibrotic signals also affect the endothelial compartment, where barrier integrity, nitric oxide bioavailability, microvascular perfusion, and paracrine communication influence whether tissue repair remains adaptive or progresses towards chronic remodelling. EndMT provides a cellular framework for examining interactions between the endothelial and interstitial compartments. However, EndMT does not necessarily represent complete conversion of endothelial cells into fibroblasts and should not be considered the predominant quantitative source of matrix-producing cells. Resident fibroblasts remain the major effector population in most forms of cardiac fibrosis [73, 74]. EndMT may instead reflect a programme of endothelial destabilisation, inflammatory amplification, and profibrotic signal propagation. Partial or transient acquisition of mesenchymal traits may therefore be relevant because it can weaken vascular identity and create conditions conducive to fibroblast activation without generating most scar-forming cells [74–77]. EndMT should be regarded as part of a broader endothelial-stromal stress response rather than as a single established driver of clinical benefit.

The stimuli associated with EndMT are heterogeneous and often coexist in metabolically stressed tissues. Glucolipotoxicity, macrophage-derived cytokines, disturbed shear stress, mechanical stretch, hypoxia, and mitochondrial ROS may disrupt endothelial quiescence and promote mesenchymal activation. In infarcted myocardium, macrophage-derived paracrine signals have been shown to promote EndMT through mechanisms involving MT1-MMP and TGF-β1, suggesting a link between inflammatory cell recruitment, endothelial plasticity, and fibrotic remodelling [78]. Metabolic factors may also contribute. Acetate availability and lactate-induced Snail1 lactylation have both been associated with EndMT and cardiac fibrosis, suggesting that local metabolic conditions may influence endothelial plasticity alongside cytokine signalling [79, 80]. Mechanical and microvascular factors may further modify this response. Experimental evidence suggests that endothelial integrin-linked kinase may limit coronary microvascular dysfunction and EndMT, whereas loss of endothelial stability is associated with microvascular rarefaction and fibrotic progression [81]. EndMT may therefore reflect the combined effects of metabolic stress, inflammatory signalling, and microvascular injury on the endothelial phenotype.

At the signalling level, TGF-β/Smad, Notch, and Wnt/β-catenin pathways interact within a profibrotic network rather than operating as independent linear cascades. TGF-β/Smad signalling is the most extensively characterised regulator of EndMT, promoting repression of endothelial markers and induction of mesenchymal genes, including α-SMA, fibronectin, and collagen-related transcripts [82]. Notch signalling can cooperate with TGF-β to sustain mesenchymal activation, whereas Wnt/β-catenin signalling contributes to endothelial plasticity, fibroblast activation, and extracellular matrix remodelling in several cardiovascular contexts [83, 84]. These interactions argue against attributing EndMT to any single canonical pathway. Endothelial plasticity may instead reflect the combined effects of inflammation, oxidative stress, substrate availability, mechanical forces, and epigenetic regulation. Partial EndMT may be reversible and adaptive during tissue repair, whereas sustained EndMT-like programming may impair endothelial barrier function, reduce capillary stability, and promote fibrosis.

Evidence linking SGLT2 inhibition to EndMT comes primarily from experimental cardiac and endothelial models. In rats with type 2 diabetes, dapagliflozin reduced cardiac fibrosis together with EndMT and fibroblast activation through AMPKα/TGF-β/Smad-related signalling [85]. In models of angiotensin II-induced cardiac remodelling and normoglycaemic chronic HF, dapagliflozin also attenuated myocardial fibrosis and reduced TGF-β1/Smad pathway activation [86, 87]. These findings are consistent with possible anti-EndMT and antifibrotic effects but do not establish that EndMT inhibition mediates clinical benefit. SGLT2 inhibitors may also reduce several upstream factors associated with endothelial destabilisation, including metabolic stress, inflammatory signalling, oxidative injury, and maladaptive TGF-β activity.

The SIRT1-Notch1 axis provides a more specific proposed link between nutrient sensing and endothelial plasticity. In experimental models, dapagliflozin reduced EndMT in association with enhanced SIRT1-mediated deacetylation of endothelial Notch1 and reduced Notch1-driven mesenchymal activation [88]. The SIRT1-Notch1 axis may therefore link cellular energy status with transcriptional and post-translational regulation. However, EndMT regulation is unlikely to depend on a single predominant pathway. The SIRT1-Notch1 mechanism should instead be regarded as one component of a broader network that includes TGF-β/Smad, Wnt/β-catenin, oxidative stress, mitochondrial injury, and inflammatory crosstalk. Accordingly, SGLT2 inhibitors may reduce EndMT by modifying several conditions that sustain mesenchymal activation rather than by acting through a single endothelial pathway.

Several endothelial effects associated with SGLT2 inhibitors are consistent with this indirect model. During cardiac ischaemia/reperfusion, dapagliflozin attenuated endothelial dysfunction and microvascular injury in association with normalisation of the XO-SERCA2-CaMKII-cofilin pathway and preservation of endothelial barrier and cytoskeletal integrity. These microvascular changes may reduce the oxidative and mechanical stimuli that favour EndMT-like endothelial activation [54]. In cardiac remodelling models, reduced Wnt/β-catenin signalling following SGLT2 inhibitor treatment further suggests that endothelial and interstitial profibrotic pathways may be affected concurrently [89]. These findings support possible effects on endothelial function but do not establish EndMT as an obligatory mediator. Changes in oxidative stress, mitochondrial quality control, or macrophage-fibroblast crosstalk could reduce fibrosis without a substantial quantitative contribution from EndMT.

**Fig. 2:**
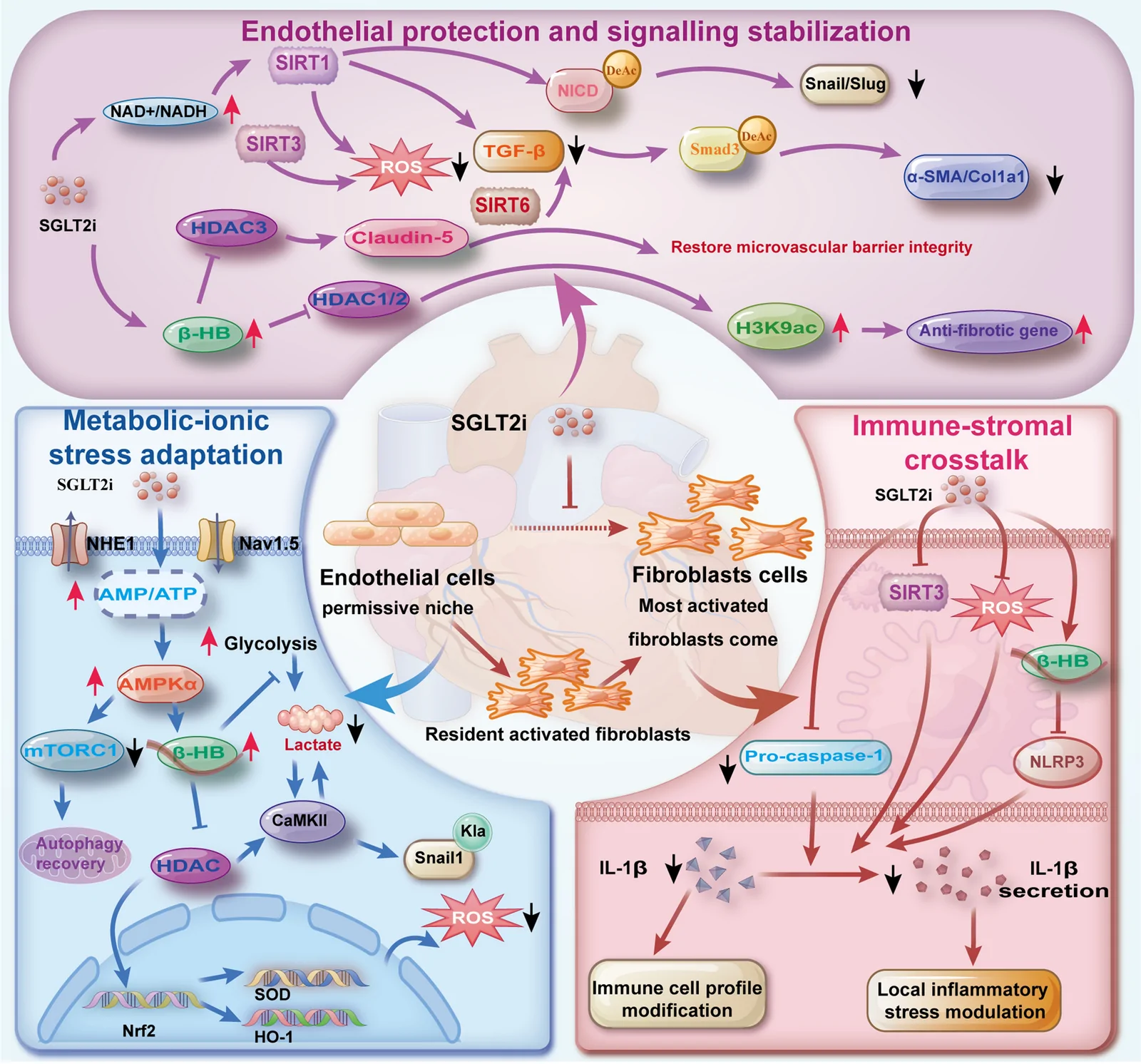
Cellular and microenvironmental pathways linking SGLT2 inhibition to endothelial protection, inflammatory modulation and EndMT-related fibrotic remodeling. SGLT2 inhibition has been linked to attenuation of microenvironmental stress through interconnected endothelial, metabolic, immune and stromal pathways. Metabolic–ionic stress adaptation involves NHE1- and Nav1.5-related sodium handling, AMP/ATP–AMPKα signalling, mTORC1 restraint and autophagy-related stress responses, which may improve ionic homeostasis, mitochondrial resilience and redox control. β-HB, lactate-related Snail1 lactylation and antioxidant signalling are shown as metabolic–epigenetic and redox-associated nodes that connect substrate stress with endothelial plasticity and ROS limitation. Endothelial protection involves NAD⁺/NADH balance, sirtuin activity, HDAC-related regulation, Notch and TGF-β/Smad3 signalling, with downstream effects on mesenchymal drivers, matrix-associated proteins and endothelial barrier integrity. Immune–stromal crosstalk is represented by β-HB- and SIRT3-associated restraint of NLRP3 inflammasome activation, reduced caspase-1 activity and lower IL-1β release, which may weaken paracrine cues that sustain resident fibroblast activation and extracellular matrix deposition. This framework links SGLT2 inhibition to maintenance of endothelial homeostasis and attenuation of adverse fibrotic remodelling, while EndMT-like endothelial plasticity remains a context-dependent component rather than a single validated mediator of clinical benefit. Abbreviations: α-SMA, α-smooth muscle actin; AMP, adenosine monophosphate; AMPKα, AMP-activated protein kinase α; ATP, adenosine triphosphate; β-HB, β-hydroxybutyrate; CaMKII, Ca^2^⁺/calmodulin-dependent protein kinase II; Col1a1, collagen type I alpha 1 chain; DeAc, deacetylation; EndMT, endothelial-to-mesenchymal transition; HDAC, histone deacetylase; HO-1, haem oxygenase 1; H3K9ac, histone H3 lysine 9 acetylation; IL-1β, interleukin-1β; KLa, lysine lactylation; mTORC1, mechanistic target of rapamycin complex 1; NAD⁺/NADH, oxidised/reduced nicotinamide adenine dinucleotide; Nav1.5, voltage-gated sodium channel 1.5; NHE1, sodium–hydrogen exchanger 1; NICD, Notch intracellular domain; NLRP3, NOD-like receptor family pyrin domain-containing 3; Nrf2, nuclear factor erythroid 2-related factor 2; ROS, reactive oxygen species; SGLT2, sodium-glucose cotransporter 2; SIRT, sirtuin; SIRT3, sirtuin 3; SOD, superoxide dismutase; TGF-β, transforming growth factor-β

An important translational limitation is the absence of direct evidence from human myocardium. Clinical imaging findings, collagen biomarkers, and HF outcomes may be consistent with reduced adverse remodelling but do not directly demonstrate suppression of EndMT in human myocardial tissue. Current human evidence cannot determine whether changes in remodelling reflect altered endothelial plasticity, reduced fibroblast activation, macrophage reprogramming, haemodynamic unloading, improved energetics, or a combination of these processes. EndMT remains relevant as a framework linking endothelial dysfunction, inflammation, and fibrosis, but its role in SGLT2 inhibitor-associated tissue protection remains context-dependent and incompletely validated. A stage-dependent effect is plausible: SGLT2 inhibitors may have greater effects on early endothelial destabilisation and inflammatory profibrotic signalling than on established microvascular loss or mature scar tissue. Further evaluation will require human tissue studies, spatial transcriptomics, lineage-informed biomarkers, and endothelial-state markers capable of distinguishing transient endothelial activation from sustained mesenchymal conversion (Fig. 2).

## Physiology and Pharmacology of SGLT2 inhibitors

The observation that SGLT2 inhibitors retain cardio-renal benefits when their glucose-lowering efficacy is attenuated suggests the involvement of mechanisms beyond antihyperglycaemic activity. Inhibition of proximal tubular glucose transport initiates a series of renal, metabolic, haemodynamic, and cellular responses that vary with tissue context and disease state. The pharmacology of SGLT2 inhibitors encompasses transporter distribution, molecular selectivity, systemic exposure, pharmacodynamic responses, and safety considerations.

### Tissue-specific expression of the SGLT family and pharmacological implications

The tissue distribution and distinct functions of SGLT family members help explain key features of gliflozin therapy. Under physiological conditions, SGLT2 is a low-affinity, high-capacity transporter located predominantly in the S1 and S2 segments of the renal proximal tubule, where it mediates most filtered glucose reabsorption [2, 90]. By contrast, SGLT1 is a high-affinity transporter that reabsorbs the remaining glucose in the S3 segment and also mediates intestinal glucose absorption. This spatial distribution allows selective SGLT2 inhibition to produce substantial glycosuria without completely blocking renal glucose reabsorption. Residual SGLT1-mediated glucose reabsorption in the S3 segment limits maximal UGE during selective SGLT2 blockade, a phenomenon commonly termed renal escape [2, 90].

Beyond the renal-intestinal axis, the pharmacological relevance of extrarenal SGLT isoforms remains incompletely defined. Cardiac SGLT1 expression has been reported to increase under pathological conditions, including ischaemia, hypertrophy, and diabetic cardiomyopathy, suggesting that SGLT1 may contribute to stress-related myocardial signalling [57, 91]. However, its contribution to overall myocardial glucose uptake remains uncertain. Recent studies in mice and humans identified a truncated SLC5A1 transcript in myocardial tissue that lacks key transmembrane domains required for sodium and glucose binding. In that study, cardiac SGLT1 appeared to make little contribution to overall myocardial glucose uptake [92]. These findings suggest that direct modulation of bulk myocardial glucose transport is unlikely to be the primary explanation for the cardiovascular benefits observed with SGLT inhibition.

SGLT expression in cardiovascular tissues may be regulated by inflammatory and metabolic stress rather than representing a fixed glucose-transport mechanism. Human myocardial studies have shown that SGLT1 is more abundant than SGLT2 in cardiac tissue and that its expression is associated with NADPH oxidase activity, inflammatory markers, and fibrosis-related gene expression [57]. More recent human vascular and cardiac data suggest that low-grade inflammation may regulate SGLT2 expression and is associated with impaired eNOS-NO signalling and increased ROS generation [93]. Together, these observations support a context-dependent interpretation of SGLT expression in cardiovascular tissues. Local SGLT expression may reflect, and in some settings contribute to, an inflammatory and oxidatively stressed microenvironment.

This distinction has implications for dual SGLT1/2 inhibition. Highly selective SGLT2 inhibitors act primarily through renal glucose handling, natriuretic and osmotic effects, and systemic metabolic adaptation [2, 90]. Additional SGLT1 blockade may extend these pharmacological effects to intestinal glucose absorption, postprandial metabolism, and selected stress-related tissue responses [90, 94]. In a mouse model of diabetic cardiomyopathy, cardiac SGLT1 was implicated in HF progression, suggesting possible pathological relevance in specific metabolic contexts [95]. Nevertheless, whether dual SGLT1/2 inhibition provides direct cardiac tissue protection beyond established renal and systemic effects remains uncertain. The rationale for dual inhibition may therefore be most relevant to selected cardiometabolic settings, while its specific effects on myocardial remodelling require further clinical and mechanistic evaluation [94, 95].

### Structural basis and selectivity of SGLT2 inhibition

The pharmacological distinction between selective SGLT2 inhibition and broader SGLT blockade begins with transporter architecture. Recent cryogenic electron microscopy studies have resolved the human SGLT2-MAP17 complex, indicating that MAP17 supports SGLT2 function partly through interactions involving transmembrane helix 13 [96, 97]. Glucose transport proceeds through a coordinated conformational cycle in which the transporter transitions from an outward-open state to a substrate-bound occluded intermediate with closed extracellular and intracellular gates and then to an inward-facing configuration that permits substrate release [98, 99]. Gliflozins inhibit this cycle by stabilising transporter conformations that are incompatible with productive glucose translocation.

The development from phlorizin to modern gliflozins reflects progressive structural refinement. Phlorizin, a naturally occurring O-glucoside, inhibits both SGLT1 and SGLT2 with limited isoform selectivity and may bind differently from modern synthetic inhibitors [97, 100]. Contemporary gliflozins use a more metabolically stable C-glycoside scaffold and a dual-binding architecture directed primarily towards the outward-facing state [101]. The glycone moiety acts as a glucose-like anchor within the central substrate-binding pocket, whereas the aglycone group extends into the extracellular vestibule and forms hydrophobic interactions with residues such as Phe98 and Phe453 [96, 101, 102]. This binding mode stabilises SGLT2 in an outward-open conformation and impedes progression to the occluded and inward-facing states required for substrate transport [99, 101].

Isoform selectivity arises largely from structural differences outside the conserved central glucose-binding pocket. The extracellular vestibule of SGLT2 accommodates bulky hydrophobic aglycone substituents, whereas SGLT1 is subject to greater steric constraints [97, 101, 103]. Structure-guided and computational studies continue to refine this model of selectivity and may inform rational modification of the aglycone region to generate agents with varying degrees of SGLT1 and SGLT2 inhibition [102–105]. The distinction between selective SGLT2 inhibition and dual SGLT1/2 inhibition therefore reflects transporter structure, although the clinical implications of broader SGLT blockade also depend on pharmacodynamic, tissue-specific, and safety considerations beyond binding affinity.

### Pharmacokinetics

The chemical modifications that enhanced SGLT2 target engagement also influenced the pharmacokinetic properties of modern gliflozins. Phlorizin established that renal glucose reabsorption could be pharmacologically inhibited, but its O-glucoside linkage was susceptible to intestinal β-glucosidase-mediated hydrolysis, making it unsuitable for sustained oral therapy [100]. Replacement of this linkage with a C-glycoside scaffold improved chemical and metabolic stability, enabling modern SGLT2 inhibitors to achieve clinically useful oral exposure and sustained target inhibition [101, 106, 107]. Although transporter binding determines selectivity, clinical use also depends on systemic stability, absorption, and clearance.

Following oral administration, SGLT2 inhibitors generally have predictable absorption and elimination profiles compatible with once-daily dosing, although individual agents differ in bioavailability, time to peak concentration, protein binding, and routes of elimination [106, 107]. Formulation may further affect absorption. A population pharmacokinetic study comparing a novel empagliflozin L-proline formulation with the conventional formulation in healthy participants found comparable systemic exposure, supporting bioequivalence while showing that formulation can alter early pharmacokinetic parameters without materially changing overall drug exposure [108]. Most agents are extensively protein bound and have elimination half-lives sufficient to maintain pharmacological activity over a 24-h dosing interval [106, 107]. These properties support once-daily administration and may facilitate adherence during long-term cardio-renal-metabolic therapy.

SGLT2 inhibitors are cleared predominantly through glucuronidation rather than extensive cytochrome P450-mediated oxidation. Hepatic and extrahepatic uridine 5′-diphospho-glucuronosyltransferase (UGT) enzymes contribute to the formation of inactive glucuronide metabolites, whereas CYP-mediated metabolism generally has a smaller role. This metabolic profile may reduce the likelihood of major CYP-mediated drug-drug interactions, which is relevant for patients with diabetes, CKD, and HF who frequently receive multiple concomitant therapies. Nevertheless, clinically relevant interactions cannot be excluded. Variations in UGT activity, transporter-mediated disposition, renal or hepatic function, and concomitant medications may influence drug exposure in selected patients [106, 107]. The overall interaction profile of SGLT2 inhibitors is therefore generally favourable but remains dependent on clinical context.

Renal impairment creates a distinct dissociation between pharmacokinetics and pharmacodynamics. As estimated glomerular filtration rate (eGFR) declines, systemic exposure to some SGLT2 inhibitors may increase to varying degrees because of changes in renal and non-renal clearance [106, 107, 109]. Conversely, the primary glucose-lowering pharmacodynamic response, UGE, decreases because less filtered glucose reaches the proximal tubule [106, 109, 110]. Model-based meta-analysis has shown that the relationships among SGLT2 inhibitor dose, UGE, and HbA1c reduction are influenced by patient characteristics, including renal function, and that UGE may serve as a pharmacodynamic reference for comparing glucose-lowering responses across agents [110]. Physiologically based pharmacokinetic/pharmacodynamic modelling of multiple SGLT2 inhibitors in renal impairment further showed that changes in drug exposure and UGE do not necessarily occur in parallel [109].

This PK/PD dissociation is relevant to the clinical interpretation of SGLT2 inhibitor therapy. Declining renal function reduces the filtered glucose load and thereby attenuates UGE and HbA1c lowering but does not eliminate systemic drug exposure or other downstream pharmacological responses. The persistence of cardio-renal benefits in patients with impaired kidney function therefore cannot be assessed solely from glycosuric efficacy. Renal function differentially affects two aspects of SGLT2 inhibitor action: the glucose-lowering response, which depends strongly on filtered glucose delivery, and broader physiological responses, including changes in renal haemodynamics, volume redistribution, and cellular stress adaptation, which may remain clinically relevant despite attenuated glycosuria. This distinction supports the assessment of pharmacodynamic effects beyond UGE.

### Pharmacodynamics

The immediate on-target consequence of SGLT2 blockade is increased UGE. This glycosuric response lowers fasting glucose and HbA1c in patients with preserved renal function but also illustrates an important clinical dissociation. Because UGE is strongly determined by the filtered glucose load, its magnitude decreases substantially as eGFR declines [109]. However, cardiovascular, renal, and HF benefits have been observed even when glycaemic efficacy is attenuated, suggesting that the pharmacodynamic effects of SGLT2 inhibitors cannot be assessed solely through glucose clearance [111–115].

Glucose remaining in the tubular lumen also produces an osmotic fluid response. In acute HF cohorts, empagliflozin increased fractional glucose excretion and plasma osmolality without a comparable increase in fractional sodium excretion or urine osmolality, suggesting that early osmotic diuresis is driven primarily by glycosuria rather than sustained natriuresis [14]. Compared with intensive loop diuresis, this profile may support decongestion with less abrupt intravascular volume contraction. This fluid response occurs alongside changes in renal haemodynamics. By increasing solute delivery from the proximal tubule to the macula densa, SGLT2 inhibitors may restore TGF and attenuate maladaptive intraglomerular hypertension. The resulting initial “dip” in eGFR is generally regarded as a functional haemodynamic response rather than evidence of acute structural kidney injury [116]. Recognising this expected early decline is clinically important because unnecessary discontinuation may reduce the longer-term renal and cardiovascular benefits observed with treatment.

These intrarenal effects are accompanied by systemic pharmacodynamic responses. Glycosuria-associated changes in proximal tubular transport may influence uric acid handling through pathways involving GLUT9 and URAT1, potentially contributing to the reduction in serum uric acid observed during SGLT2 inhibitor therapy [117, 118]. A recent multicenter retrospective cohort study provided further evidence of this uric acid-lowering effect, particularly in patients with baseline hyperuricaemia and HF [119]. Haematological changes are also observed. Mediation analyses from EMPA-REG OUTCOME identified increases in haematocrit and haemoglobin and reductions in uric acid as variables strongly associated with lower cardiovascular mortality during empagliflozin treatment [120]. These associations do not establish that erythropoiesis alone accounts for the survival benefit but suggest that haematological adaptation may contribute to the broader treatment response. Mechanistic and clinical studies further suggest that the increase in haematocrit is not solely a passive consequence of plasma volume contraction, as empagliflozin may induce early changes in erythropoietin, iron utilisation, red blood cell indices, and related erythropoietic markers [30, 121].

Although this profile, including glycosuria, osmotic fluid regulation, reduced intraglomerular pressure, uric acid lowering, and haematological adaptation, is broadly shared across the drug class, complete interchangeability should not be assumed. Real-world comparative-effectiveness data suggest that cardiovascular effectiveness is generally similar among individual SGLT2 inhibitors at clinically effective doses, whereas comparisons involving low or non-equivalent exposures may yield differences in specific endpoints, such as HF hospitalisation [122]. The clinical expression of SGLT2 inhibitor pharmacodynamics therefore reflects both class-wide mechanisms and drug-specific exposure–response thresholds.

### Physiological consequences of SGLT2 inhibition

Glycosuria produces a sustained osmotic effect that may preferentially reduce extracellular or interstitial fluid excess. Compared with the abrupt intravascular volume depletion that can occur with conventional loop diuresis, this response may relieve congestion with less evidence of severe intravascular contraction or reflex neurohormonal activation. Clinical findings are consistent with this compartmentalised effect. In CKD, empagliflozin has been associated with reductions in fluid overload, body weight, and blood pressure. However, an EMPA-KIDNEY mechanistic MRI substudy found no clear evidence of renal or cardiac structural remodelling after 18 months, suggesting that systemic physiological effects may not correspond directly to detectable imaging changes [16, 123]. In HFpEF, dapagliflozin reduced plasma volume and lowered resting and exercise pulmonary capillary wedge pressure [34]. In an exploratory study of patients with type 2 diabetes and established cardiovascular disease or high cardiovascular risk, 12 weeks of empagliflozin lowered cardiac filling pressures without an observed reflex increase in sympathetic activity [124]. Together, these findings suggest that the renal osmotic effects of SGLT2 inhibition may contribute to lower congestion burden, reduced filling pressure, and less mechanical stress on the failing heart. Glycosuria also produces ongoing caloric loss and may modify systemic nutrient sensing.

The resulting reduction in the insulin-to-glucagon ratio may shift systemic metabolism towards a fasting-like state characterised by lipid mobilisation and mild ketogenesis [29, 36]. Experimental models initially suggested that this metabolic shift might provide the failing myocardium with a more efficient alternative substrate. However, the EMPA-VISION trial found no significant improvement in the myocardial phosphocreatine-to-ATP ratio during empagliflozin treatment in human HF cohorts [44]. Similarly, experimental findings of altered substrate utilisation and improved mitochondrial redox status in failing rat hearts should not be assumed to represent consistent enhancement of myocardial energy production in humans [53]. Mechanistic analyses from the EMPEROR programme further suggest a complex erythropoietic and iron-mobilisation response, with empagliflozin treatment associated with activation of the erythropoietin-erythroferrone-TfR1 axis, increased iron utilisation, and higher haemoglobin levels in patients with HF [125]. These findings support a more cautious interpretation: the cardiovascular and renal benefits observed may be associated with broader adaptation to nutrient- and oxygen-sensing stress rather than a direct ketone-mediated increase in ATP production alone.

The associated reduction in nutrient-surplus signalling may shift cellular activity from anabolic processes towards stress adaptation and cellular maintenance. Haematological changes provide one measurable component of this response. Increases in haematocrit and haemoglobin appear to involve erythropoietin signalling, altered iron handling, and changes in red blood cell indices rather than haemoconcentration alone [30–32]. At the vascular and tissue levels, SGLT2 inhibition has been associated with changes in arterial stiffness, endothelial function, and mitochondrial oxidative stress, although these effects vary among study populations and vascular endpoints [126, 127]. Experimental studies also suggest greater cellular tolerance of ionic, oxidative, and mitochondrial stress, including preservation of mitochondrial quality control and endothelial barrier stability under ischaemic or toxic conditions [48, 54–56]. These effects may attenuate the metabolic and inflammatory stress associated with maladaptive remodelling, although causal relationships with regression of human fibrosis or EndMT-specific clinical benefit remain unclear. Overall, SGLT2 inhibition may influence cardio-renal-metabolic physiology through a combination of modest decongestion, altered substrate availability, haematological adaptation, changes in vascular function, and modulation of cellular stress responses.

### Preclinical safety pharmacology

Empagliflozin underwent dedicated cardiovascular, respiratory, and central nervous system safety assessments during nonclinical development. Single-dose studies found no effects on the cardiovascular endpoints assessed in telemetered dogs or on respiratory and neurobehavioural endpoints in rats. In isolated guinea-pig papillary muscle, action potential configuration and contractile function remained unchanged at concentrations up to 10 μmol/L [128]. These findings apply to specific experimental conditions and do not establish comparable safety margins for other SGLT2 inhibitors.

Complementary repeated-dose toxicology studies reported dehydration, electrolyte disturbances, and renal histopathological changes that varied by dose and species. Much of this toxicity was attributed to exaggerated pharmacological effects associated with urinary glucose loss [128]. Interpretation of these findings requires consideration of systemic exposure, species susceptibility, and the distinction between functional and histopathological endpoints.

The developing kidney presents an additional safety consideration. A systematic review of animal studies and regulatory data reported renal pelvic and tubular dilatation following exposure to several SGLT2 inhibitors during renal maturation. Reversibility after treatment withdrawal varied among studies, with incomplete recovery reported for canagliflozin, dapagliflozin, and ertugliflozin [129]. These observations raise concern about exposure during kidney development but do not permit direct safety comparisons among agents or determine renal risk in treated adults.

## Clinical applications of SGLT2 inhibitors

**Fig. 3:**
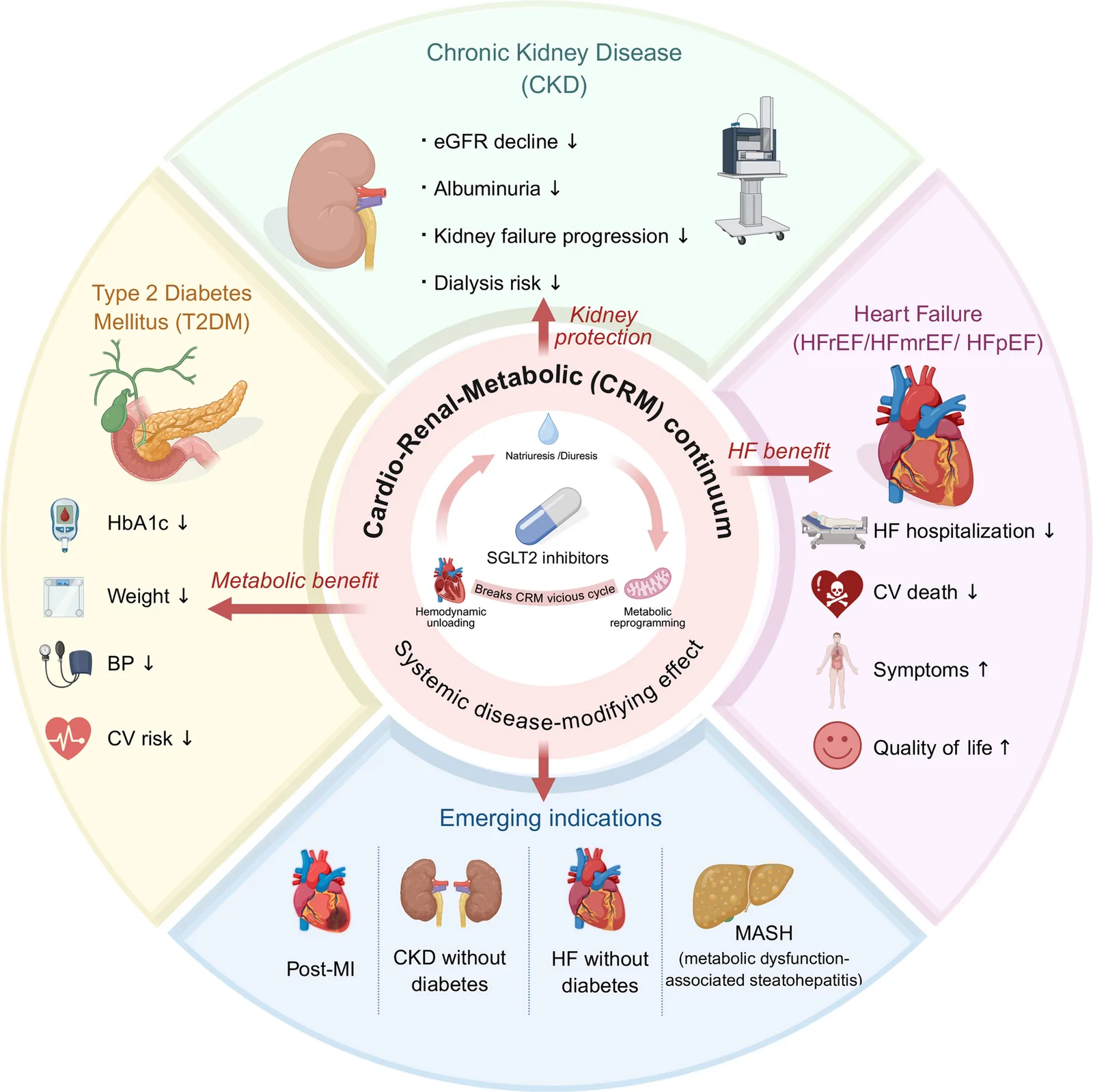
Clinical efficacy of SGLT2 inhibitors across the cardio-renal-metabolic continuum. SGLT2 inhibitors are positioned at the centre of the cardio-renal-metabolic (CRM) continuum, with natriuresis or diuresis, haemodynamic unloading and metabolic reprogramming shown as core disease-modifying axes. Metabolic benefit: In type 2 diabetes mellitus (T2DM), SGLT2 inhibition is associated with reductions in HbA1c, body weight and blood pressure (BP), together with cardiovascular (CV) risk modification. Kidney protection: In chronic kidney disease (CKD), treatment slows estimated glomerular filtration rate (eGFR) decline, reduces albuminuria and delays progression towards kidney failure or dialysis. Heart failure benefit: Across HFrEF, HFmrEF and HFpEF, treatment reduces heart failure hospitalisations or worsening heart failure events and improves symptoms and quality of life; effects on CV death vary across populations and trials. Emerging and expanding indications: Non-diabetic CKD and heart failure are established extensions of clinical benefit, whereas post-myocardial infarction (MI) risk and metabolic dysfunction-associated steatohepatitis (MASH) remain areas of ongoing investigation. Abbreviations: HbA1c, glycated haemoglobin; HFmrEF, heart failure with mildly reduced ejection fraction; HFpEF, heart failure with preserved ejection fraction; HFrEF, heart failure with reduced ejection fraction; SGLT2, sodium-glucose cotransporter 2

Clinical evidence for SGLT2 inhibitors has broadened from glucose lowering in type 2 diabetes to improved outcomes in CKD and HF. Evidence was established through disease-specific trials, and the benefits observed across several populations suggest that shared cardio-renal-metabolic mechanisms may contribute. The most consistent clinical findings are reductions in HF hospitalisation and kidney disease progression, whereas cardiovascular mortality benefits are most apparent in selected high-risk populations (Fig. 3).

### Type 2 diabetes mellitus (T2DM)

SGLT2 inhibitors were initially developed as glucose-lowering agents. By inhibiting proximal tubular glucose transport, they induce glycosuria and reduce glycated haemoglobin (HbA1c) in T2DM [1]. This mechanism reduces hyperglycaemia with a lower intrinsic risk of hypoglycaemia than insulin secretagogues and is accompanied by modest, generally consistent reductions in body weight and systemic blood pressure [3]. These effects are relevant to the complex comorbidity profile of T2DM. However, experience with older glucose-lowering agents showed that improving these surrogate markers alone did not fully address residual cardiovascular mortality risk.

Cardiovascular outcome trials (CVOTs) expanded the clinical role of SGLT2 inhibitors beyond glucose lowering. EMPA-REG OUTCOME provided early evidence of cardiovascular benefit beyond HbA1c reduction, reporting a 38% relative reduction in cardiovascular mortality among high-risk patients with T2DM [111]. Subsequent findings from the CANVAS Program [114], DECLARE-TIMI 58 [115], and VERTIS CV [130] broadened the evidence across diabetic populations. Although the magnitude of benefit for major adverse cardiovascular events (MACE) and cardiovascular mortality varied among trials, reductions in HF hospitalisation and favourable renal outcomes were more consistent [114, 115, 130]. The early separation of HF-related event curves suggested the involvement of rapidly acting mechanisms because this timing is unlikely to be explained by the gradual regression of atherosclerotic plaques.

This temporal pattern supports a distinction between cardiovascular outcomes and glucose-lowering efficacy. Real-world comparative-effectiveness studies found that SGLT2 inhibitor use, compared with dipeptidyl peptidase-4 (DPP-4) inhibitor use, was associated with lower risks of HF and acute kidney injury (AKI) across diverse T2DM populations and baseline HbA1c levels [131]. Multinational observational cohorts, including CVD-REAL 2, similarly reported associations with lower mortality and cardiovascular event rates in routine clinical practice [132]. Mediation analyses from EMPA-REG OUTCOME identified changes in haematocrit, haemoglobin, and uric acid as statistical mediators of the reduction in cardiovascular mortality with empagliflozin [120]. These systemic changes have been interpreted within a proposed pseudo-starvation model, in which sustained caloric loss may modify energy-sensing and survival pathways in the stressed myocardium [37, 133].

As the evidence developed, clinical use extended to higher-risk T2DM populations. The SCORED trial reported clinical benefits in patients with T2DM and advanced CKD, while SOLOIST-WHF reported fewer subsequent cardiovascular events when treatment was initiated around an episode of worsening HF [134, 135]. Both trials evaluated sotagliflozin, a dual SGLT1/2 inhibitor, and should therefore be interpreted as evidence for the broader strategy of sodium–glucose cotransporter inhibition rather than selective SGLT2 inhibition alone. These findings have contributed to updates in international diabetes management guidelines. Contemporary T2DM management increasingly considers cardiovascular and kidney risk alongside glycaemic control. In this context, SGLT2 inhibitors, often considered alongside glucagon-like peptide-1 (GLP-1) receptor agonists, support treatment strategies that address cardio-renal risk in addition to reducing HbA1c.

### Chronic kidney disease (CKD)

The benefits observed in diabetic kidney disease provided a basis for ongoing evaluation in broader CKD populations. Before these trials, the management of CKD in diabetes relied primarily on metabolic control and strict blood pressure regulation. The CREDENCE trial broadened this framework by reporting a 30% reduction in the primary composite outcome among patients with diabetic kidney disease [136]. Post hoc analyses further suggested that sustained reductions in albuminuria and slower eGFR decline were not primarily explained by baseline HbA1c or subsequent improvements in systemic glycaemia [137]. This dissociation suggests that the benefits observed at lower filtration rates may partly reflect localised changes in renal haemodynamics, biomechanical stress, and metabolism. By reducing proximal tubular sodium and glucose reabsorption, SGLT2 inhibitors increase solute delivery to the macula densa and may restore TGF [2, 10]. These changes in renal haemodynamics may contribute to lower intraglomerular pressure and slower kidney disease progression, although their precise cellular contribution to antifibrotic remodelling remains incompletely defined.

Because these effects may occur partly independently of systemic glycaemic control, SGLT2 inhibitors have also been evaluated in non-diabetic CKD. The DAPA-CKD and EMPA-KIDNEY trials provided evidence for their use across diverse non-diabetic CKD populations [112, 138]. Prespecified analyses from DAPA-CKD showed reductions in major adverse kidney and cardiovascular events regardless of baseline diabetes status [139]. Evidence has also emerged in selected primary glomerular diseases. Among patients with IgA nephropathy, dapagliflozin reduced urinary albumin excretion and delayed disease progression [140]. The benefits observed also span a range of baseline albuminuria levels. Although patients with severe macroalbuminuria may experience larger absolute risk reductions because of their higher baseline risk, secondary analyses from EMPA-KIDNEY suggest that empagliflozin slows chronic eGFR decline even in patients with low levels of albuminuria [141].

The clinical benefits observed are accompanied by an early functional reduction in glomerular filtration followed by slower long-term loss of kidney function. Initiation of SGLT2 inhibition is commonly followed by an early functional “dip” in eGFR. This decline is generally interpreted as a consequence of reduced intraglomerular pressure rather than structural injury [2, 10]. Trajectory analyses showed that, after the initial decline, eGFR decreased more slowly with dapagliflozin than with placebo, supporting longer-term preservation of kidney function [142]. Early reductions in urinary albumin excretion are also associated with subsequent kidney outcomes and may provide a clinically useful marker of treatment response [143]. Nevertheless, clinicians may remain hesitant to initiate treatment in advanced kidney disease or in older and clinically vulnerable populations. Analyses of patients with stage 4 CKD (eGFR < 30 mL/min/1.73 m^2^) reported sustained effects on eGFR decline without evidence of a disproportionate increase in safety concerns [144]. Evidence in older patients with CKD similarly supports careful individualised use rather than exclusion based solely on age [145]. These randomised trial findings are consistent with large real-world datasets. The Japan CKD Database reported lower renal event rates and slower kidney function decline among patients receiving SGLT2 inhibitors than among those receiving other glucose-lowering therapies in routine clinical practice [146].

SGLT2 inhibitors are increasingly incorporated earlier in CKD management as kidney-protective therapy rather than being reserved for late complications. Long-term follow-up has reported sustained cardio-renal benefits across broad CKD populations, including patients with advanced renal impairment [147]. Together, the observed effects on renal haemodynamic, metabolic, and inflammatory stress responses support the role of SGLT2 inhibitors in contemporary cardio-renal-metabolic care.

### Heart failure (HF)

The clinical classification of HF relies substantially on left ventricular ejection fraction, which continues to guide treatment decisions. Trials of SGLT2 inhibitors have reported clinical benefits across HFrEF, HFmrEF, and HFpEF [113, 148–151]. These benefits suggest that shared cardio-renal-metabolic mechanisms may contribute to outcomes across ejection fraction categories, with potential effects on both progressive structural decline and acute decompensation [9, 113, 135]. Modulation of metabolic and microenvironmental stressors may therefore complement therapies that primarily target neurohormonal pathways [113, 149].

#### HF with reduced ejection fraction

Evidence supporting SGLT2 inhibitor use in HFrEF is based principally on the DAPA-HF and EMPEROR-Reduced trials [148, 152]. Pooled analyses of these trials support reductions in worsening HF events and a favourable effect on cardiovascular mortality [149]. Unlike therapies that often require gradual dose titration, SGLT2 inhibitors can be initiated at fixed doses and have been associated with early separation of clinical event curves, sometimes within weeks of treatment initiation [148, 152, 153]. This early clinical stabilisation, including fewer urgent hospital interventions and sustained improvements in Kansas City Cardiomyopathy Questionnaire scores, suggests that relatively rapid metabolic, haemodynamic, or cellular stress-response mechanisms may contribute in addition to delayed structural remodelling [153, 154]. This early onset is clinically relevant during periods when the risk of acute decompensation is high [9, 135, 153].

The benefits observed were broadly similar in patients with and without diabetes [155]. This pattern suggests that the clinical effects in HFrEF are not primarily dependent on systemic glucose lowering but may involve broader effects on cardio-renal haemodynamics, metabolic stress, and myocardial function [149, 155]. Renal outcomes provide complementary evidence. In DAPA-HF, dapagliflozin slowed the long-term decline in eGFR across different degrees of baseline renal impairment [156]. Concurrent cardiac and renal effects may attenuate the cardio-renal feedback cycle through which declining filtration contributes to cardiac stress via volume overload, uraemic stress, and secondary neurohormonal activation [156]. Preservation of renal function during SGLT2 inhibitor treatment may therefore represent both a parallel clinical benefit and a potential contributor to sustained HF stabilisation [149, 156].

CMR studies in HFrEF and related high-risk cardiometabolic populations, including SUGAR-DM-HF, EMPA-HEART CardioLink-6, and EMPA-TROPISM, reported reductions in left ventricular end-systolic and end-diastolic volumes and left ventricular mass [157–159]. However, volumetric indices cannot establish cellular-level antifibrotic activity. These geometric changes are consistent with reduced adverse macroscopic remodelling but cannot determine whether the structural changes reflect reduced myocyte hypertrophy, altered interstitial fluid compartmentalisation, or suppression of profibrotic programmes such as EndMT. Macroscopic imaging therefore documents structural changes but does not resolve the underlying cellular mechanisms in the human heart [157–159]. Further studies integrating clinical imaging with specific molecular biomarkers, tissue-level measurements, or spatial multi-omics approaches are needed to determine how macroscopic reverse remodelling relates to microenvironmental cellular plasticity.

#### HF with mildly reduced or preserved ejection fraction

HFmrEF and HFpEF have historically had limited treatment options. These phenotypes are associated with complex pathophysiology involving endothelial inflammation, interstitial matrix deposition, impaired ventricular relaxation, and diastolic stiffness and have shown limited and inconsistent responses to conventional neurohormonal therapies [150, 151, 160]. The EMPEROR-Preserved and DELIVER trials provided outcome evidence in this population, reporting that empagliflozin and dapagliflozin reduced the composite risk of cardiovascular death or worsening HF across the higher ejection fraction spectrum [150, 151]. These findings suggest that the limited efficacy of previous neurohormonal therapies in HFpEF may partly reflect the contribution of disease mechanisms beyond neurohormonal overactivation [113, 160]. In many HFpEF phenotypes, comorbidity-related systemic inflammation, endothelial dysfunction, reduced nitric oxide bioavailability, and microvascular impairment are thought to contribute to diastolic dysfunction [76, 160]. SGLT2 inhibitors may therefore provide benefit partly through broader metabolic, haemodynamic, and inflammatory effects relevant to microvascular dysfunction and HF progression rather than through isolated receptor blockade [113, 160].

The benefits observed are not limited to delaying a first worsening HF event. A prespecified analysis of DELIVER showed a reduction in the total burden of recurrent HF events [161]. This endpoint is particularly relevant in HFpEF, which is frequently associated with repeated hospital admissions and progressive functional decline. Assessment of total events captures cumulative disease burden rather than only the time to the first event [161]. Patient-reported health status also improved. The PRESERVED-HF trial, the EMPEROR-Preserved health-status analysis, and the remote, patient-centred CHIEF-HF study reported early and sustained improvements in Kansas City Cardiomyopathy Questionnaire (KCCQ) scores across diverse clinical populations [162–164]. Although CHIEF-HF was not restricted to HFpEF, its patient-centred design provides supportive evidence of symptomatic effects across the HF spectrum [163]. Together, these findings suggest benefits in reducing recurrent HF burden and improving patient-reported functional status [161–164].

The benefits observed in HFmrEF and HFpEF should not be directly attributed to reversal of established myocardial fibrosis, as this interpretation is not supported by the available biological evidence. Endothelial dysfunction and profibrotic signalling have been implicated in HFpEF [74, 160]. However, attenuation of upstream inflammatory and metabolic factors associated with these programmes is not equivalent to rapid enzymatic degradation of mature, cross-linked collagen [74, 77]. Clinical stabilisation may instead be associated with systemic metabolic and haemodynamic effects that reduce conditions favouring continued microvascular stress [113, 160]. Potential effects include reductions in endothelial inflammation, interstitial congestion, and metabolic strain, which may limit the persistence of a profibrotic microenvironment and signalling associated with EndMT and fibroblast activation. Current evidence therefore supports a more cautious interpretation: the benefits observed may reflect preservation of vascular function, improvement in congestion-related physiology, and slower adverse remodelling rather than direct resolution of established scar tissue [74, 76, 77, 160].

#### Acute HF and early initiation

SGLT2 inhibitors have been evaluated both in stable outpatients and during hospitalisation for acute decompensated HF. EMPULSE showed that initiating empagliflozin during hospitalisation for acute HF was feasible and generally safe and was associated with early clinical benefit [9]. SOLOIST-WHF, which evaluated the dual SGLT1/2 inhibitor sotagliflozin in patients with type 2 diabetes and recent worsening HF, further suggested that treatment initiated before or shortly after discharge may reduce early HF-related events and the total hospitalisation burden [135, 165, 166]. Because structural antifibrotic remodelling would be expected to require several months, these early clinical findings are more consistent with rapid decongestive, haemodynamic, renal, and metabolic effects than with structural remodelling alone [9, 10, 14]. The early separation of clinical trajectories may therefore reflect improved congestion control, changes in renal haemodynamics, and greater physiological stability after discharge rather than immediate reversal of myocardial fibrosis [9, 10, 14].

Acute haemodynamic responses vary among patient phenotypes. Recent studies, including DICTATE-AHF and DAPA ACT HF-TIMI 68, supported the feasibility and overall safety of early dapagliflozin initiation, although their primary decongestive and early cardiovascular endpoints were not uniformly positive [167, 168]. One possible explanation for these mixed findings is the distinct physiological profile of SGLT2 inhibitors. Unlike loop diuretics, which primarily produce sodium-rich diuresis and may rapidly reduce intravascular volume, SGLT2 inhibitors appear to generate a more electrolyte-sparing osmotic diuretic response that may preferentially affect interstitial congestion and fluid compartmentalisation [10, 14]. Conventional measures of acute volume removal, such as weight-adjusted diuretic efficiency, may therefore not fully capture these compartmentalised fluid shifts [10, 167]. Acute treatment responses may vary according to patient phenotype, baseline congestion pattern, renal function, previous diuretic exposure, and the timing of treatment initiation [167, 169].

Early initiation during the vulnerable post-discharge period should therefore be regarded as a strategy to improve clinical stability and facilitate the implementation of guideline-directed medical therapy rather than as an acute rescue intervention comparable to intravenous inotropes or high-intensity loop diuretics [9, 167, 168]. Potential benefits depend on appropriate timing and individualised clinical assessment. Treatment initiation should account for dynamic variables, including haemodynamic stability, volume status, and kidney function [169]. In this context, SGLT2 inhibitors may support the transition from acute stabilisation to chronic disease-modifying treatment, while requiring appropriate patient selection and safety monitoring [9, 167–169].

### Other potential and emerging applications

SGLT2 inhibitors are under ongoing evaluation following acute myocardial infarction (MI), but the evidence remains less developed than that for chronic HF or CKD. In EMPACT-MI, empagliflozin did not significantly reduce the primary composite endpoint of first hospitalisation for HF or death from any cause after acute MI, although subsequent analyses suggested reductions in first and total HF hospitalisations [170, 171]. These clinical findings differ from experimental models, in which empagliflozin and dapagliflozin have been associated with reduced infarct size, preserved microvascular integrity, and activation of mitochondrial quality-control pathways under controlled ischaemia–reperfusion conditions [39, 54, 56]. This discrepancy may reflect differences in ischaemic exposure and treatment timing. Experimental protocols frequently involve pretreatment or immediate administration during transient ischaemia, whereas treatment after clinical acute MI usually begins after coronary occlusion and the initiation of necrotic and inflammatory processes. Although SGLT2 inhibitors may modulate microvascular stress and substrate utilisation, as suggested by post-infarction changes in β-hydroxybutyrate dynamics, they would not be expected to reverse established acute necrotic injury or replace immediate reperfusion therapy [40]. Effects on arrhythmogenic substrates may partly reflect secondary haemodynamic and metabolic changes. Reported reductions in atrial fibrillation risk may be associated with reduced atrial mechanical stretch, lower left ventricular filling pressures, and improved decongestion, although these associations appear to vary according to the underlying clinical phenotype [172]. Experimental studies also suggest that modulation of specific non-coding RNA networks, such as the miR-181b/Sema3A axis, can attenuate TGF-β-induced endothelial plasticity and atrial fibrosis, although this evidence is not specific to SGLT2 inhibition [173]. Direct electrophysiological effects have been reported in experimental models and isolated cardiomyocytes [50–52], but their contribution to clinical outcomes remains uncertain. Modulation of inflammatory and fibrotic signalling may also influence arrhythmogenic substrate formation [68]. For example, dapagliflozin reduced cardiac fibrosis and inflammation by modulating HIF-2α signalling in a mouse model of arrhythmogenic cardiomyopathy, suggesting that SGLT2 inhibitors may influence arrhythmogenic substrate formation through metabolic and inflammatory remodelling rather than direct antiarrhythmic blockade [68]. In metabolic dysfunction-associated steatohepatitis (MASH), structural tissue changes can be quantified histologically more directly than in the myocardium. A recent randomised trial reported that dapagliflozin improved histological endpoints in biopsy-confirmed MASH, including MASH improvement without worsening of fibrosis, MASH resolution without worsening of fibrosis, and fibrosis improvement without worsening of MASH [174]. The sustained caloric loss associated with SGLT2 inhibition may promote lipid oxidation and autophagic lipid-droplet clearance, potentially reducing hepatic lipotoxic stress [47]. These liver findings suggest that SGLT2 inhibitor-associated metabolic changes may be accompanied by measurable structural improvement in a metabolically driven fibrotic disease. However, they should not be extrapolated directly to the human myocardium, where structural remodelling is more difficult to attribute to specific cellular mechanisms. In models of type 3 cardio-renal syndrome, empagliflozin has also been associated with mitochondrial quality control and microvascular protection, suggesting that some emerging applications may depend on organ-specific vascular responses rather than a uniform antifibrotic mechanism [175, 176].

Combined intestinal SGLT1 and renal SGLT2 inhibition may further modify postprandial metabolism and gut incretin signalling. Sotagliflozin has reduced worsening HF events and total HF hospitalisations in patients with diabetes and recent worsening HF and may also reduce broader cardiovascular events in selected high-risk populations [134, 135, 165, 166, 177]. Whether these outcomes arise from intestinal SGLT1 blockade, localised inhibition of stress-induced myocardial SGLT1, broader systemic metabolic effects, or combined renal–intestinal mechanisms remains uncertain. Experimental studies suggest that sotagliflozin may improve myocardial perfusion, metabolic and redox remodelling, and post-MI cardiac dysfunction, but these findings require further clinical validation [178–180]. Determining which metabolic and fibrotic responses are attributable to selective SGLT2 inhibition or dual SGLT1/2 inhibition may require detailed phenotyping and machine-learning-based clustering to identify patient subgroups more likely to benefit from each pharmacological strategy [181] (Table 1).

**Table 1:** Landmark clinical trials shaping the therapeutic role of SGLT2 inhibitors across the cardio-renal-metabolic continuum

Trial	Disease context/population	Drug/intervention	Key finding	Clinical implication	Ref
EMPA-REG OUTCOME; CANVAS Program; DECLARE-TIMI 58; VERTIS CV	T2DM with established or elevated cardiovascular risk	Empagliflozin; canagliflozin; dapagliflozin; ertugliflozin	CVOTs broadened outcome evidence beyond HbA1c lowering, with the most consistent signals for HF hospitalisation and renal protection	Repositioned SGLT2 inhibitors as cardio-renal risk-modifying therapies in T2DM	[111, 114, 115, 130]
CREDENCE	T2DM with diabetic kidney disease	Canagliflozin	Reduced the primary composite outcome and slowed kidney-function decline beyond glycaemic improvement	Established SGLT2 inhibition as kidney-protective therapy in diabetic kidney disease	[136, 137]
DAPA-CKD; EMPA-KIDNEY	CKD with or without T2DM	Dapagliflozin; empagliflozin	Reduced major kidney and cardiovascular outcomes across broad CKD populations, including non-diabetic CKD	Expanded SGLT2 inhibitor benefit beyond diabetic nephropathy	[112, 138, 139, 141, 147]
DAPA-HF; EMPEROR-Reduced	HFrEF, with or without diabetes	Dapagliflozin; empagliflozin	Reduced worsening HF events, with benefits largely independent of baseline diabetes status	Validated SGLT2 inhibitors as foundational therapy for HFrEF	[148, 149, 152, 155, 156]
EMPEROR-Preserved; DELIVER	HFmrEF or HFpEF	Empagliflozin; dapagliflozin	Reduced the composite risk of cardiovascular death or worsening HF; DELIVER also reduced recurrent HF events	Extended therapeutic benefit to the higher ejection fraction spectrum	[150, 151, 161]
EMPULSE; SOLOIST-WHF	Hospitalised acute HF or recent worsening HF	Empagliflozin; sotagliflozin	Supported early initiation during or shortly after acute/worsening HF	Supports treatment during vulnerable acute or post-discharge phases; SOLOIST-WHF represents dual SGLT1/2 inhibition evidence	[9, 135, 165, 166]
SCORED	T2DM with CKD	Sotagliflozin	Reduced cardiovascular events in diabetic CKD using dual SGLT1/2 inhibition	Supports broader sodium-glucose cotransport inhibition while remaining distinct from selective SGLT2 inhibitor evidence	[134]
EMPACT-MI	After acute myocardial infarction with elevated HF risk	Empagliflozin	Did not significantly reduce the primary composite of first HF hospitalisation or all-cause death, although HF hospitalisation signals were observed	Indicates that post-MI use remains an emerging indication	[170, 171]

*Abbreviations*: *CKD* chronic kidney disease, *CVOTs* cardiovascular outcome trials, *HbA1c* glycated haemoglobin, *HF* heart failure, *HFmrEF* heart failure with mildly reduced ejection fraction, *HFpEF* heart failure with preserved ejection fraction, *HFrEF* heart failure with reduced ejection fraction, *MI* myocardial infarction, *SGLT1/2* sodium-glucose cotransporters 1 and 2, *SGLT2* sodium-glucose cotransporter 2, *T2DM* type 2 diabetes mellitus

### Clinical safety and risk management

As the clinical use of SGLT2 inhibitors broadens, their safety profile remains closely related to their renal, metabolic, and haemodynamic effects. Several recognised adverse events are associated with these pharmacological effects. SGLT2 inhibition may lower the threshold for diabetic ketoacidosis (DKA) under susceptible conditions. Recognition can be difficult because SGLT2 inhibitor-associated DKA may occur without marked hyperglycaemia despite substantial metabolic acidosis [182–184]. Although meta-analyses of large cardiovascular outcome trials suggest that DKA is infrequent in patients with type 2 diabetes [185], this mechanism-related risk requires careful perioperative management, particularly during prolonged fasting, infection, major surgery, severe physiological stress, or acute insulin withdrawal [182, 186]. Perioperative observational studies provide additional evidence regarding postoperative outcomes but do not establish the optimal duration of treatment interruption [187, 188].

Persistent glycosuria also alters the genitourinary microenvironment and is associated with an increased risk of genital mycotic infections [131]. However, large comparative-effectiveness studies distinguish this localised mycotic risk from severe urinary tract infections, for which no consistent increase has been demonstrated [189]. These studies also suggest a more favourable renal safety profile than initially anticipated. Compared with DPP-4 inhibitors, SGLT2 inhibitor use has been associated with a lower incidence of AKI in patients with type 2 diabetes, despite higher rates of genital infections and DKA [131].

These findings do not suggest a general increase in AKI risk but do not eliminate the need for careful assessment of volume status. Volume depletion, hypotension, and worsening kidney function remain important considerations, particularly in patients with HF receiving intensive background diuretic therapy. Although glycosuria-induced osmotic diuresis may reduce effective circulating volume in susceptible patients, a modest early decrease in estimated glomerular filtration rate in a clinically stable patient may reflect expected changes in renal haemodynamics rather than structural nephrotoxicity [116]. Progressive deterioration in kidney function or a decline accompanied by hypotension or hypovolaemia should not be assumed to represent this expected response [186]. Misinterpreting the functional haemodynamic dip as AKI may prompt unnecessary treatment discontinuation and reduce the longer-term benefits observed. A trial meta-analysis and a population-based CKD cohort also reported a lower risk of AKI with SGLT2 inhibitors in patients with type 2 diabetes [190, 191].

The amputation signal observed in the CANVAS programme has not been consistently reproduced in other trials [114, 186]. Nationwide registry data suggested possible increases in amputation and DKA compared with GLP-1 receptor agonists [192]. In a separate cohort of patients with CKD and type 2 diabetes, SGLT2 inhibitor use was associated with higher risks of lower-limb amputation and non-vertebral fracture than GLP-1 receptor agonist use [190]. Conversely, a cohort study of patients with a relatively low baseline fracture risk found no increase in fracture risk with canagliflozin compared with GLP-1 receptor agonists [193]. These heterogeneous findings require consideration of differences in study populations, comparator treatments, and baseline risk and do not establish a uniform safety profile across agents or patient groups.

Risk management should therefore be based on context-specific assessment rather than broad avoidance. In patients with CKD and those receiving multiple concomitant therapies, particular attention should be given to baseline volume status, kidney function trajectory, susceptibility to infection, and concurrent medications [186]. A history of DKA, peripheral vascular disease, or diabetic foot complications should also be considered [182, 186]. These considerations apply both to established indications and to ongoing evaluation in emerging clinical settings (Table 2).

**Table 2:** Pharmacological features, safety concerns, and clinical management considerations of SGLT2 inhibitors

Pharmacological effect/feature	Potential adverse event or clinical concern	Clinical interpretation/management	Ref
Selective SGLT2 blockade vs. dual SGLT1/2 inhibition	Distinct gastrointestinal and metabolic profiles	Interpret clinical efficacy and safety according to transporter selectivity; dual SGLT1/2 inhibition may produce additional intestinal and postprandial metabolic effects and should not be treated as fully equivalent to selective SGLT2 blockade	[2, 90, 94, 95]
Sustained glycosuria and altered genitourinary microenvironment	Genital mycotic infections; concern about urinary tract infections	Genital mycotic infections are a consistent safety signal, whereas a consistent increase in severe urinary tract infections has not been demonstrated. Provide hygiene education, early symptom recognition and prompt treatment in susceptible patients	[131, 189]
Osmotic diuresis and altered fluid handling	Volume depletion, hypotension or dizziness, particularly in patients receiving intensive diuretic therapy	Assess volume status, blood pressure and background diuretic use before and during therapy; adjust concomitant loop diuretics or antihypertensive agents when clinically needed	[14, 24, 186]
Metabolic shift and pseudo-starvation signalling	Euglycaemic diabetic ketoacidosis	Apply sick-day guidance and perioperative precautions. Temporarily withhold treatment during prolonged fasting, surgery or critical illness, and assess promptly for ketoacidosis when clinically suspected	[182, 184, 186]
TGF restoration	Early functional decline in eGFR misclassified as AKI	Interpret a modest early eGFR dip as a functional reduction in intraglomerular pressure when clinically stable; monitor the renal trajectory rather than discontinuing therapy prematurely	[17–19, 116]
Pharmacodynamic dissociation at low eGFR	Reduced UGE and attenuated HbA1c lowering	Recognise that cardio-renal benefits may persist despite weakened glycaemic efficacy; continuation should be based on organ-protective indication, tolerability and renal-function thresholds	[106, 109, 112, 113, 186]
Erythropoietic and uric-acid-lowering responses	Misinterpretation as simple haemoconcentration or isolated urate therapy	Interpret haematocrit rise and uric acid reduction as systemic pharmacodynamic signatures	[30, 31, 117, 118, 121]
Context-dependent safety in CKD, HF, older or frail patients	AKI concern, frailty-related intolerance, polypharmacy issues, and historical amputation or fracture signals	Available evidence does not suggest an increased AKI risk, whereas findings on amputation and fracture risk are less consistent; individualise treatment according to volume status, kidney-function trajectory, diabetic foot risk, vascular disease, frailty and concomitant medications	[186, 190–193]

*Abbreviations*: *AKI* acute kidney injury, *CKD* chronic kidney disease, *eGFR* estimated glomerular filtration rate, *HbA1c* glycated haemoglobin, *HF* heart failure, *SGLT1/2* sodium-glucose cotransporters 1 and 2, *SGLT2* sodium-glucose cotransporter 2, *TGF* tubuloglomerular feedback, *UGE* urinary glucose excretion

## Translational challenges and future applications

The growing clinical use and ongoing evaluation of SGLT2 inhibitors raise a central translational challenge: linking observed clinical benefits to specific tissue-level mechanisms in heterogeneous patient populations. Although their use has broadened across the cardio-renal-metabolic continuum, direct evidence explaining their tissue-level effects in humans remains incomplete. Event-based outcomes, renal function trajectories, and imaging-derived remodelling markers have documented clinical benefits in several disease settings but cannot identify the cellular processes associated with treatment response in individual patients. Further progress will require closer integration of circulating biomarkers, tissue-level molecular data, imaging phenotypes, and patient-stratification methods to relate clinical responses more precisely to underlying biological mechanisms.

### Gaps between clinical benefit and mechanistic attribution

Clinical trials have shown that SGLT2 inhibitors reduce worsening HF events and slow kidney function decline across broad populations, but these outcomes do not identify the responsible tissue pathways [138, 149, 150, 152]. Imaging studies have reported reductions in left ventricular mass, chamber volumes, and ECV following SGLT2 inhibitor treatment [157, 158, 194, 195]. These findings are compatible with reduced adverse remodelling but are not specific to any single cellular mechanism. Changes in these indices may reflect altered loading conditions, reduced myocyte hypertrophy, interstitial fluid redistribution, changes in collagen turnover, or attenuation of fibrosis. Without cell-state-specific measurements, these possibilities cannot be distinguished with sufficient confidence.

This limitation is particularly relevant to endothelial plasticity-related mechanisms, including EndMT, because current human evidence cannot reliably distinguish endothelial state transitions from related processes such as endothelial activation, inflammation, and matrix remodelling. Preclinical studies combining endothelial lineage tracing with single-cell analysis can distinguish changes in endothelial fate from transient mesenchymal activation with a resolution not currently achievable in human myocardium [196]. Human cardiovascular tissue studies have identified endothelial heterogeneity and mesenchymal-associated features, including in calcific aortic valve disease [197, 198]. However, these observations do not establish endothelial fate conversion in human myocardium. EndMT-related signatures may support an association between endothelial injury and fibrotic remodelling but cannot establish that the clinical benefits of SGLT2 inhibition are mediated by suppression of EndMT. The principal challenge is to distinguish EndMT from endothelial activation, fibroblast expansion, immune-cell recruitment, and matrix turnover. This distinction is necessary to avoid interpreting favourable clinical or imaging outcomes as direct evidence of a specific cellular pathway.

### Biomarkers, imaging, and multi-omics approaches

Conventional imaging and circulating biomarkers remain important components of mechanistic studies but provide contextual rather than cell-state-specific information. CMR-derived ECV, left ventricular mass index (LVMi), and chamber volumes provide measures of myocardial tissue characteristics and cardiac remodelling [157, 158, 194]. Circulating inflammatory markers and collagen-turnover peptides provide complementary information on systemic inflammation and matrix turnover [62, 63, 71]. However, these measurements cannot determine whether structural changes reflect altered loading conditions, interstitial fluid redistribution, reduced myocyte hypertrophy, collagen remodelling, or endothelial cell-state transitions [195, 199]. Future studies may therefore use these measures as clinically interpretable anchors alongside more specific molecular assessments.

Single-cell RNA sequencing (scRNA-seq) and spatial transcriptomics allow the cellular composition and spatial organisation of the fibrotic niche to be examined. These approaches may distinguish activated fibroblast states, endothelial activation, partial mesenchymal-like endothelial states, and macrophage–endothelial crosstalk within the same tissue context [196, 198, 200]. This distinction is particularly important in human myocardium, where genetic lineage tracing cannot be performed and endothelial activation may overlap with transient EndMT-like marker expression. Spatially resolved datasets may also determine whether candidate endothelial or stromal signatures are localised to areas of microvascular rarefaction, inflamed interstitium, or extensive matrix deposition. However, most human datasets are cross-sectional and cannot replace dynamic fate mapping. They should therefore be used to strengthen mechanistic associations and improve cell-state resolution rather than to claim definitive evidence of endothelial fate transition.

Metabolomic, proteomic, and epigenetic profiling may further relate the systemic pharmacology of SGLT2 inhibitors to local tissue biology. Candidate measurements include acetate availability, lactate-induced Snail1 lactylation, mitochondrial calcium signalling, ketone-related metabolic adaptation, and nutrient-sensing pathways [35, 79, 80, 201]. These markers should be used not merely to restate known metabolic effects of SGLT2 inhibition but to assess whether systemic metabolic changes are associated with local endothelial stress, inflammatory signalling, matrix turnover, or EndMT-related endothelial features. Future studies could combine imaging, circulating biomarkers, and spatial molecular profiling in phenotype-guided cohorts, particularly in heterogeneous conditions such as HFpEF, CKD, and diabetes-associated cardiac disease [176, 181]. Such designs may clarify how clinical responses to SGLT2 inhibitors relate to specific cellular mechanisms, including endothelial plasticity-related processes, while reducing reliance on clinical endpoints or imaging surrogates alone for mechanistic inference.

### Patient stratification and precision medicine

The benefits observed across the ejection fraction spectrum and irrespective of baseline diabetes status suggest that traditional disease classifications may not fully explain heterogeneity in treatment response [202, 203]. Analysing these agents uniformly across heterogeneous syndromes such as HFpEF may obscure differences in the clinical and biological contexts associated with benefit [181, 203]. Stratification may therefore extend beyond fixed functional measures, such as left ventricular ejection fraction or diabetes status, to include cardio-renal-metabolic overlap, kidney function trajectory, frailty, body mass index, and patient-reported health status [203–207]. These variables may help identify subgroups in whom responses to SGLT2 inhibitors are associated primarily with decongestion, changes in renal haemodynamics, metabolic adaptation, or attenuation of inflammatory and microvascular stress.

The stage of tissue remodelling may also influence treatment response. Transient endothelial mesenchymal activation has been observed during post-infarction repair [196], and SGLT2 inhibitors can affect myofibroblast differentiation in cultured human cardiac fibroblasts [208]. Whether these effects are associated with greater clinical benefit during early remodelling than after established scar formation remains uncertain. This distinction is important because favourable outcomes or improved imaging markers do not necessarily indicate structural reversal of established fibrosis. Precision-medicine approaches will require integration of machine-learning-based clinical clustering with tissue-informed molecular profiling, including single-cell and spatially resolved methods. These strategies may identify subgroups characterised by active microvascular inflammation, endothelial injury, or metabolic–fibrotic coupling rather than predominantly established scar tissue [181, 200].

### Selective SGLT2 inhibition versus dual SGLT1/2 inhibition

Precision phenotyping also requires clearer differentiation between selective SGLT2 blockade and dual SGLT1/2 inhibition [91]. Selective SGLT2 inhibitors act primarily through renal glucose and sodium handling, whereas dual inhibitors such as sotagliflozin extend transporter inhibition to the gastrointestinal tract. Additional intestinal SGLT1 inhibition can delay postprandial glucose absorption and modify incretin-related responses, including glucagon-like peptide-1 (GLP-1) signalling [91, 209]. These pharmacodynamic differences suggest that selective SGLT2 inhibition and dual SGLT1/2 inhibition should not be assumed to be interchangeable.

Clinical outcome data for sotagliflozin have shown reductions in worsening HF events and the total hospitalisation burden among patients with type 2 diabetes and recent worsening HF [166]. In patients with type 2 diabetes, CKD, and additional cardiovascular risk, a prespecified analysis of SCORED also suggested reductions in major adverse cardiovascular events, including myocardial infarction and stroke [177]. However, the mechanisms underlying this possible ischaemic benefit remain uncertain. Potential contributors include intestinal SGLT1 blockade, renal SGLT2 inhibition, systemic metabolic and incretin-related effects, changes in myocardial perfusion and redox remodelling observed in preclinical chronic ischaemia models, and gut microbiome-related mechanisms reported in experimental myocardial infarction models [91, 178, 180, 209]. Dual inhibition should therefore be regarded as a pharmacologically distinct strategy that may be relevant to selected metabolic, cardio-renal, or ischaemic-risk phenotypes rather than as a universal substitute for selective SGLT2 inhibition [177, 209].

### Combination therapies and future clinical trial design

Pathological tissue remodelling involves overlapping and interacting signalling networks, including TGF-β-, Notch-, and Wnt-related pathways, which may limit the effects of interventions targeting a single microenvironmental pathway [76]. Future combination strategies should therefore be based on complementary biological mechanisms rather than the accumulation of therapies. SGLT2 inhibitors have established cardio-renal-metabolic effects through changes in renal haemodynamics, decongestion, and metabolic responses. However, patients with advanced tissue remodelling, persistent inflammation, or high residual cardio-renal risk may require additional interventions targeting incretin signalling, mineralocorticoid pathways, neurohormonal activation, inflammation, or fibrotic remodelling [210–212].

Current evidence supports further evaluation of combination strategies, although its strength varies among combinations. SMART-C analyses suggest that the benefits of SGLT2 inhibitors are generally similar among patients with diabetes with or without background GLP-1 receptor agonist therapy, supporting potential combined use in selected patients with metabolic risk [211]. Model-based analyses further suggest that combining SGLT2 inhibitors, GLP-1 receptor agonists, and non-steroidal mineralocorticoid receptor antagonists may provide clinically relevant improvements in cardiovascular, kidney, and survival outcomes among patients with type 2 diabetes and albuminuria [210]. The CONFIDENCE trial provided more direct evidence that initial combination treatment with finerenone and empagliflozin produced a greater reduction in the urinary albumin-to-creatinine ratio than either treatment alone in patients with CKD and type 2 diabetes [212]. These findings suggest additive or complementary therapeutic effects but do not establish biological synergy across all outcomes.

Future clinical trials should extend beyond efficacy-only assessment towards mechanism-informed evaluation. Event-driven endpoints remain essential but should be accompanied by dynamic functional measures, including early eGFR trajectories, albuminuria responses, congestion markers, patient-reported outcomes, and prespecified safety monitoring [207]. Circulating biomarkers and single-cell or spatially resolved molecular profiling may help associate treatment responses with tissue-level pathways involving endothelial stress, inflammatory activation, matrix turnover, and metabolic remodelling [200]. Target trial emulation and other real-world causal-inference methods may complement randomised trials when direct head-to-head or long-term combination trials are impractical, although residual confounding must still be considered [213]. Such designs may clarify whether a pharmacological combination improves clinical outcomes and identify the patient subgroups and tissue-level pathways associated with treatment response (Table 3).

**Table 3:** Translational roadmap for mechanism-informed precision use of SGLT2 inhibitors

Translational gap	Proposed solution	Expected value	Ref
Mechanistic attribution: Clinical endpoints and imaging markers lack cell-state specificity	Single-cell profiling, spatial transcriptomics and molecular profiling	Help distinguish endothelial activation, EndMT-like endothelial states, fibroblast activation and tissue remodelling pathways	[195, 196, 198–200]
Patient stratification: Ejection fraction, diabetes status and eGFR incompletely capture treatment-response heterogeneity	Clinical clustering combined with biomarkers, metabolomics and congestion phenotyping	Identify candidate patient subgroups characterised by microvascular inflammation, metabolic stress or congestion-sensitive physiology	[181, 202–205]
Pharmacological specificity: Selective SGLT2 inhibition and dual SGLT1/2 inhibition are not interchangeable	Phenotype-guided evaluation of intestinal, postprandial and metabolic effects	Test whether selective or dual inhibition should be prioritised in specific metabolic, intestinal or ischaemic-risk phenotypes	[91, 177, 209]
Combination therapy: Residual inflammation, fibrosis, obesity-related HFpEF and albuminuric CKD may persist	Mechanism-informed trials with GLP-1 receptor agonists, mineralocorticoid receptor antagonists, renin–angiotensin system blockers or targeted anti-inflammatory therapies	Guide rational sequencing and combination therapy beyond single-agent treatment	[210–213]

*Abbreviations*: *CKD* chronic kidney disease, *eGFR* estimated glomerular filtration rate, *EndMT* endothelial-to-mesenchymal transition, *GLP-1* glucagon-like peptide 1, *HFpEF* heart failure with preserved ejection fraction, *SGLT1/2* sodium-glucose cotransporters 1 and 2, *SGLT2* sodium-glucose cotransporter 2

## Conclusion and perspectives

SGLT2 inhibitors have progressed from glucose-lowering agents that induce glycosuria to therapies associated with clinical benefits across the cardio-renal-metabolic continuum. This broader clinical use suggests that inhibition of proximal tubular transport may influence disease-related processes through renal, haemodynamic, metabolic and cellular stress responses. Benefits have been observed particularly in conditions involving intraglomerular hypertension, congestion, metabolic inflexibility, chronic low-grade inflammation and microvascular dysfunction. However, these agents should not be regarded as universal antifibrotic or anti-inflammatory therapies. Their effects may instead reflect modulation of interacting processes involving renal transport, fluid distribution, nutrient sensing, mitochondrial function, inflammation and tissue remodelling.

An important unresolved issue is the relationship between clinical benefit and tissue-level mechanisms. Outcome trials have shown reductions in HF events and kidney disease progression, but the cellular effects of SGLT2 inhibitors in the human myocardium and kidney remain incompletely characterised. Imaging indices, circulating biomarkers and event-based endpoints can document clinical benefits but cannot determine whether the effects observed are associated with haemodynamic unloading, interstitial fluid redistribution, cardiomyocyte remodelling, reduced fibroblast activation, macrophage reprogramming or changes in endothelial cell states. This limitation is particularly relevant to EndMT-related mechanisms. Experimental studies suggest that preservation of endothelial homeostasis and attenuation of EndMT-like signalling may contribute to tissue protection, but direct human evidence is insufficient to establish EndMT suppression as a necessary mediator of the benefits of SGLT2 inhibitors. Further research should therefore determine which endothelial, stromal and immune-cell states are modified in human disease and whether these changes are causal, adaptive or secondary markers of broader improvements in the tissue microenvironment.

A further research priority is to define the temporal boundary between potentially reversible stress-related remodelling and established structural damage. Early endothelial activation, inflammatory microvascular dysfunction and metabolically associated matrix turnover may be more responsive to SGLT2 inhibition, whereas advanced collagen cross-linking, capillary rarefaction and terminal fibroblast activation may be less reversible. Future studies should determine when endothelial stress develops into a stable fibrotic microenvironment, identify biomarkers that distinguish active matrix turnover from established scar and clarify why rapid decongestion is not always accompanied by structural reverse remodelling. They should also assess whether earlier initiation of SGLT2 inhibitors can limit irreversible tissue remodelling more effectively than later treatment of established disease. Addressing these questions will require longitudinal sampling, serial imaging and molecular phenotyping rather than assessment at a single time point.

Future clinical studies should also move beyond broad disease categories towards mechanism-informed stratification. Diabetes status, ejection fraction and estimated glomerular filtration rate remain important clinical variables, but they do not fully account for heterogeneity in responses to SGLT2 inhibitors. More informative stratification may incorporate congestion phenotype, kidney function trajectory, cardio-renal-metabolic overlap, frailty, body composition, inflammatory burden, metabolomic profile and patient-reported health status. Spatial transcriptomics, single-cell profiling and proteomic or metabolomic signatures could help determine whether a patient’s disease is characterised predominantly by decongestion-responsive physiology, renal haemodynamic stress, inflammatory endothelial activation, metabolic inflexibility or active fibrotic turnover. Such mechanistic endotyping may also clarify whether selective SGLT2 inhibition is sufficient in particular populations or whether dual SGLT1/2 inhibition may be more relevant in selected metabolic, intestinal–incretin or ischaemic-risk phenotypes.

Combination therapy represents another priority for future research and should be guided by complementary biological mechanisms rather than the accumulation of treatments alone. SGLT2 inhibitors may provide a basis for cardio-renal-metabolic therapy, but patients with persistent inflammatory activation, advanced tissue remodelling, obesity-related HFpEF, high albuminuric risk or residual metabolic dysfunction may require additional interventions. Combinations with GLP-1 receptor agonists, mineralocorticoid receptor antagonists, renin–angiotensin system blockers or targeted anti-inflammatory and antifibrotic therapies should be evaluated for their effects on distinct but interacting pathways. Future outcome trials should therefore incorporate mechanistic substudies that include dynamic measures of kidney function and congestion, circulating biomarkers, non-invasive molecular imaging, spatial multi-omics and prespecified safety monitoring. Target-trial emulation and adaptive platform designs may complement randomised trials when long-term or multi-arm combination studies are difficult to conduct. Future research should determine how and when SGLT2 inhibitors should be used, which patients are most likely to benefit and which therapeutic combinations provide complementary clinical and biological effects.

## Data Availability

Not applicable.
